# CTCF’s loop-independent functions prevail over chromatin looping in the acute degradation system

**DOI:** 10.1093/procel/pwaf087

**Published:** 2025-11-04

**Authors:** Gongcheng Hu, Binrui Ji, Jie Zhang, Yanjiang Liu, Yuli Lu, Xiuqin Wang, Huawei Ren, Junzhi Liao, Hongjie Yao

**Affiliations:** Division of Life Sciences and Medicine, University of Science and Technology of China, Hefei 230026, China; Department of Basic Research, Guangzhou National Laboratory, Guangzhou 510005, China; Division of Life Sciences and Medicine, University of Science and Technology of China, Hefei 230026, China; Guangzhou Institutes of Biomedicine and Health, Chinese Academy of Sciences, Guangzhou 51030, China; Department of Basic Research, Guangzhou National Laboratory, Guangzhou 510005, China; Guangzhou Institutes of Biomedicine and Health, Chinese Academy of Sciences, Guangzhou 51030, China; Division of Life Sciences and Medicine, University of Science and Technology of China, Hefei 230026, China; Department of Basic Research, Guangzhou National Laboratory, Guangzhou 510005, China; Department of Basic Research, Guangzhou National Laboratory, Guangzhou 510005, China; Department of Basic Research, Guangzhou National Laboratory, Guangzhou 510005, China; Guangzhou Institutes of Biomedicine and Health, Chinese Academy of Sciences, Guangzhou 51030, China

**Dear Editor,**

Trans-regulatory elements mediate long-range chromatin interactions, essential for gene expression regulation. The CCCTC-binding factor (CTCF), a versatile DNA-binding protein, collaborates with cohesin to pause cohesin extrusion and establish relatively stable chromatin loops. CTCF-mediated chromatin loops (commonly termed CTCF loops) exhibit dual roles. These architectural elements facilitate transcriptional activation by spatially approximating distal enhancers with target promoters (Popay and Dixon, 2022). Conversely, they also block enhancer–­promoter (EP) interactions to repress gene expression (Song et al., 2022). These opposing roles underscore CTCF’s importance in maintaining the balance between gene activation and repression, ultimately influencing cell function and development.

Recent investigations employing acute protein degradation systems coupled with chromatin conformation capture techniques like Hi-C and Micro-C assays have provided new insights into CTCF’s role in genome organization. While acute CTCF depletion significantly compromises higher-order chromatin organization—including disruption of topologically associating domains (TADs) and chromatin loops—its impact on EP interactions and transcriptional output appears remarkably modest (Hsieh et al., 2022; Nora et al., 2017). The precise molecular mechanisms underlying this apparent dichotomy remain to be fully elucidated.

To elucidate CTCF’s gene regulatory roles, we employed a mouse embryonic stem cell (mESC) line harboring CTCF fused to the auxin-induced degron (AID) system (Nora et al., 2017), referred to as CTCF^AID^, maintained in medium containing two inhibitors (PD0325901 and CHIR99021). Robust CTCF degradation was confirmed by both Western blot analysis and chromatin immunoprecipitation followed by sequencing (ChIP-seq) following indole-3-acetic acid (IAA) treatment (Fig. S1A and S1B). Our RNA-seq data revealed that a large number of genes were differentially expressed after 12 h of IAA treatment (Fig. S1C). Extended depletion (24 h) resulted in an increased number of differentially expressed genes (DEGs) (Fig. S1D), while maintaining expression patterns consistent with the 12-h time point (Fig. S1E).

Comparative analysis with published CTCF degradation datasets across multiple mESC lines (Hsieh et al., 2022; Kubo et al., 2021; Nora et al., 2017) revealed that only three genes were consistently co-upregulated (Fig. S1F), while 36 genes were consistently downregulated (Fig. 1A). Intriguingly, all co-downregulated genes exhibited robust CTCF occupancy at their promoters (Fig. 1B). These downregulated genes are likely positively regulated by CTCF, potentially through loop-dependent mechanisms. From this cohort, *Ppa2* and *Zbtb39* were chosen for detailed study due to their pronounced transcriptional downregulation upon CTCF depletion, strong CTCF occupancy at their promoters, and the presence of downstream super-enhancers (SEs) (Figs. 1C, S1G and S2A).

**Figure 1.**
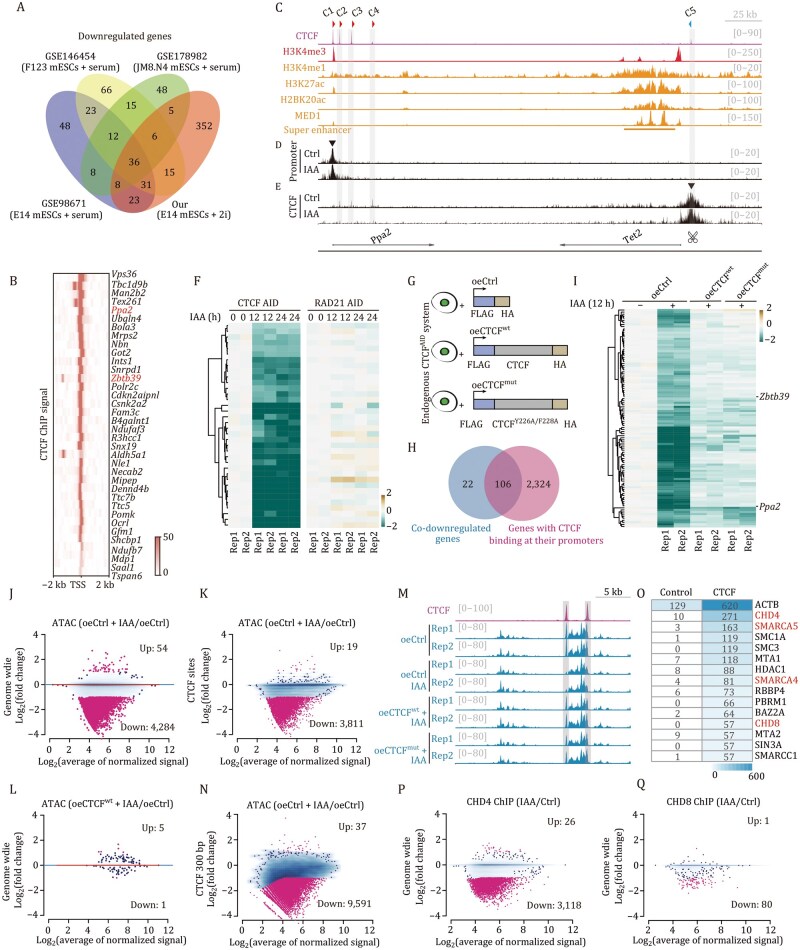
**Promoter-bound CTCF enhances chromatin accessibility through the promotion of CHD4 and CHD8 binding in a loop-­independent manner**. (A) Overlap analysis of downregulated genes following 24 h of CTCF degradation across our and previously published RNA-seq datasets. (B) Heatmap showing CTCF ChIP enrichment at promoters of co-downregulated genes from panel (A). (C) Genomic tracks depicting CTCF signal, active histone markers, and super-enhancer at *Ppa2* locus (chr3: 133,300,000–133,600,000). (D and E) M4C tracks illustrating chromatin interactions mediated by *Ppa2* promoter (panel D) or CTCF C5 site (panel E) in CTCF^AID^ mESCs with (12 h) or without IAA treatment. (F) Heatmaps showing expression patterns of co-downregulated genes from panel B in RNA-seq datasets following CTCF or RAD21 degradation. (G) Experimental design for rescue assays: control, exogenous expression of CTCF^wt^ or CTCF^mut^ in CTCF^AID^ mESCs. (H) Venn diagram showing overlap between co-downregulated genes from Fig. S4H and genes with promoter-proximal CTCF binding. (I) Expression heatmap of the overlapped genes from panel (H). (J) Scatter plot depicting the changes of genome-wide ATAC signals in untreated and IAA-treated CTCF-AID mESCs. (K) Scatter plot showing the changes of ATAC signals at CTCF-overlapping regions in untreated and IAA-treated CTCF-AID mESCs. The total number of CTCF peaks (29,934) is listed in Table S6. (L) Genome-wide ATAC signal comparison between control and oeCTCF^wt^ plus IAA-treated CTCF^AID^ mESCs. (M) Genomic tracks of ATAC signal changes at chr9: 40,315,000–40,340,000 upon different treatments. (N) CTCF-centered (300 bp windows) ATAC signal changes between untreated and IAA-treated CTCF^AID^ mESCs. (O) Top 15 CTCF potential interacting proteins identified by ChIP-MS from Fig. S7A. (P and Q) Genome-wide binding changes of CHD4 (panel P) or CHD8 (panel Q) in untreated and IAA-treated CTCF^AID^ mESCs.

The *Ppa2* promoter region harbors four forward-oriented CTCF binding sites (C1 in the proximal promoter and C2–C4 downstream) (Fig. 1C). These sites may engage in chromatin looping with the reverse-oriented C5 site adjacent to the SE, potentially facilitating EP interaction to boost *Ppa2* expression. To investigate these potential interactions, we developed a high-resolution chromatin capture assay called MNase-based 4C (M4C) (Fig. S3A), which achieves high resolution of chromatin interactions (Fig. S3B) and reliably detects diverse chromatin inter­action patterns independent of primer orientation (Fig. S3C–H). Surprisingly, neither significant EP interactions nor CTCF loops were detected at the *Ppa2* promoter (Fig. 1D). However, M4C analysis targeting the C5 site revealed interactions with downstream CTCF sites (C2–C4) but not with promoter–proximal C1 site (Fig. 1E). While these C5-mediated interactions could potentially facilitate spatial proximity between the C5 site and *Ppa2* promoter to promote gene expression, CRISPR/Cas9-mediated deletion of the C5 site had no significant impact on *Ppa2* expression (Fig. S1H–J), suggesting that *Ppa2* regulation is independent of CTCF loops.

Similarly, *Zbtb39*, which exhibited comparable CTCF binding and enhancer patterns, showed no interaction between its promoter and the downstream SE (Fig. S2A and S2B). Deletion of the C6 CTCF site, which demonstrated interaction with the *Zbtb39* promoter in M4C assays (Fig. S2C), had no effect on gene expression (Fig. S2D–F). Collectively, these findings demonstrate that CTCF regulates both *Ppa2* and *Zbtb39* expression through mechanisms independent of chromatin looping.

To confirm the loop-independent nature of this regulation, we acutely degraded RAD21 (Fig. S2G and S2H), a key cohesin subunit involved in loop extrusion (Li et al., 2020). RAD21 depletion did not alter the expression of any of the 36 CTCF-dependent downregulated genes (shown in Fig. 1A), including *Ppa2* and *Zbtb39* (Fig. 1F), supporting the hypothesis that these genes are not regulated by CTCF loops.

To further assess the loop-independent function of CTCF, we utilized a CTCF Y226A/F228A double mutation system, which disrupts CTCF loops without affecting CTCF binding (Li et al., 2020). We overexpressed either wild-type CTCF (oeCTCF^wt^) or Y226A/F228A-mutated CTCF (oeCTCF^mut^) in CTCF^AID^ cells (Figs. 1G and S4A). M4C and Hi-C assays confirmed that oeCTCF^wt^ could but not oeCTCF^mut^ restored CTCF loops after degradation (Fig. S4B–D). RNA-seq analysis revealed that the mRNA expression level of exogenous CTCF was slightly higher than its endogenous level (Fig. S4E), and neither oeCTCF^wt^ nor oeCTCF^mut^ significantly impacted gene expression (Fig. S4F). Certain IAA-responsive genes consistently downregulated or upregulated in all IAA-treated cells (Fig. S4G) were excluded. Further analysis of the co-downregulated genes in CTCF-degraded cells (Fig. S4H) revealed that 82.8% (106/128) exhibited significant CTCF occupancy at their promoter regions (Fig. 1H). Compared with the expression pattern of downregulated genes without CTCF binding in their promoters (Fig. S4I), transcriptional repression of most CTCF-bound genes could be rescued by ­overexpression of either CTCF^wt^ or CTCF^mut^ (Fig. 1I), indicating that these genes are regulated by a CTCF loop-independent mechanism. These results strongly support a model wherein CTCF positively regulates gene expression through mechanisms independent of chromatin loops.

CTCF has been reported to upregulate gene expression by suppressing antisense RNA transcription at divergent promoters (Luan et al., 2022). Our TT-seq data corroborate this finding, showing decreased expression of CTCF target genes following acute CTCF degradation (Fig. S5A). Notably, this transcriptional reduction was partially reversed by either oeCTCF^wt^ or oeCTCF^mut^ (Fig. S5B), reinforcing CTCF’s direct role in gene transcription regulation. Consistent with the prior report, we detected elevated antisense RNA levels at CTCF target loci upon CTCF degradation (Fig. S5C). However, systematic analysis revealed that only a minority of CTCF-regulated genes showed ­significant antisense transcription (Fig. S5D), indicating that while antisense suppression contributes to CTCF-­mediated regulation at some loci, the predominant mechanism of CTCF-dependent transcriptional activation likely involves other, currently unidentified loop-independent processes.

Previous studies have shown that acute CTCF depletion leads to comparable numbers of increased and decreased chromatin-accessible sites (Xu et al., 2021), with a subset colocalizing with CTCF, indicating the existence of indirect regulation. To specifically examine CTCF's direct role in modulating chromatin accessibility, we performed ATAC-seq on CTCF^AID^ cells with and without IAA treatment. Analysis of nucleosome-free regions revealed a striking asymmetry in CTCF-dependent chromatin accessibility changes: while only 54 sites showed increased accessibility, 4,284 sites exhibited significant reductions following CTCF depletion (Fig. 1J). Notably, about 90% (3811/4284) of downregulated accessible sites overlapped with CTCF ChIP-seq peaks (Figs. 1K and S6A), the remaining 10% downregulated accessible sites (Group 2) also exhibited obvious CTCF binding signals (Fig. S6A), which were not identified as significant peaks by MACS2 using default parameter. But motif analysis revealed that about 85% of Group 2 sites indeed contain CTCF motifs, albeit with ­relatively weak motif strength compared with those in Group 1 (Fig. S6B and S6C). Importantly, both CTCF^wt^ and CTCF^mut^ were able to rescue CTCF loss-mediated downregulation of chromatin accessibility (Figs. 1L and S6D), providing compelling evidence that CTCF directly maintains chromatin accessibility.

To investigate the relationship between reduced chromatin accessibility with transcriptional changes following CTCF degradation, gene annotation was performed for decreased accessible sites (from Fig. 1J). The results showed that only 4% of these sites were located within promoter regions, corresponding to 143 genes (Fig. S6E). Of these genes, the expression of 71 genes was not detected in our RNA-seq data. Among the remaining 72 expressed genes, only 19 genes showed reduced expression following CTCF degradation (Fig. S6F and S6G).

To elucidate the mechanisms underlying the discordance between reduced promoter accessibility and stable gene expression, several potential explanations were considered based on the following observations. In the case of *Smpd4*, a CTCF binding site is located within its promoter region (−1000 to +1000 bp relative to transcription start site, TSS) but outside the core promoter region. Upon CTCF depletion, chromatin accessibility decreased at the CTCF-bound site without affecting accessibility at the core promoter, and *Smpd4* expression stably remained (Fig. S6H). Additionally, the *Dlg3* gene utilizes multiple alternative promoters, with CTCF bound to only one of them. CTCF degradation led to reduced accessibility specifically at this alternative promoter, but overall *Dlg3* expression was not significantly altered (Fig. S6I).

Our results revealed that less than 20 genes exhibited downregulation despite a significant decrease of chromatin accessibility at their promoters. However, over 100 genes with CTCF binding at their promoters were downregulated upon CTCF degradation in the RNA-seq data (Fig. 1H and 1I). Detailed analysis showed that the overall chromatin accessibility at their promoters showed no significant changes, but there was a clear reduction in accessibility specifically at CTCF binding sites (Fig. S6J–L). Further analysis revealed that CTCF depletion preferentially reduced accessibility at sharply defined genomic regions (Fig. 1M). Genome-wide analysis of 300 bp windows centered on CTCF binding sites identified an even more substantial reduction of accessibility, with 9,591 sub-regions showing significant downregulation (Fig. 1N). These findings suggest that CTCF primarily affects chromatin accessibility in regions tightly localized to its binding sites. In addition, analysis of downregulated genes revealed that the center of CTCF motifs is positioned approximately 57 bp upstream of the TSSs (Fig. S6M), consistent with previous findings (Nora et al., 2017), which underscores the functional importance of precise CTCF positioning at ­promoters in facilitating gene expression.

CTCF depletion caused decreased chromatin accessibility; however, only a subset of CTCF-bound sites showed accessibility reduction upon depletion (Fig. 1K and 1N), suggesting context-dependent regulatory mechanisms. Previous studies have linked CTCF to several chromatin remodeling complexes, including (i) SMARCA5, which facilitates CTCF binding (Bomber et al., 2023; Song et al., 2022) and (ii) CHD8, reported to interact with CTCF ­(Ishihara et al., 2006). To systematically investigate whether CTCF regulates chromatin accessibility by modulating chromatin remodeler binding, we analyzed CTCF ChIP-MS data from mESCs (Ortabozkoyun et al., 2022). This revealed CTCF association with core subunits of major remodeling complexes: CHD4 and CHD8 (NuRD/CHD complexes), SMARCA5 (ISWI complex), and SMARCA4/BRG1 (SWI/SNF complex) (Figs. 1O and S7A). Transcription analysis showed high expression of *Chd4*, *Smarca5*, and *Smarca4* in mESCs, while *Chd8* level is relatively low (Fig. S7B). Co-immunoprecipitation experiments validated physical interactions between CTCF and CHD4, BRG1, and SMARCA5 (Fig. S7C). Since the detection of CHD8 was challenging using commercial antibodies, we constructed endogenous biotin-tagged *Chd8* mESC lines (Fig. S7D). However, unlike previous reports (Ishihara et al., 2006), we failed to detect stable CTCF–CHD8 interaction (Fig. S7E), potentially due to the transient nature of this interaction or the low expression of *Chd8* in mESCs.

To investigate whether CTCF regulates chromatin accessibility through influencing the binding of chromatin remodelers, we performed ChIP-seq for CHD4, CHD8, BRG1, and SMARCA5 in CTCF^AID^ mESCs. Since commercial antibodies failed to work for ChIP, we established endogenously biotin-tagged mESC lines for each factor, validated by Western blot (Fig. S7D), and generated high-quality, reproducible biotin ChIP-seq datasets (Fig. S7F and S7G). Our results revealed that CHD4 binding showed pronounced reduction following CTCF depletion (Fig. 1P), while CHD8 binding exhibited modest but significant decrease (Fig. 1Q). Notably, more than half of downregulated CHD4 sites and nearly all of downregulated CHD8 sites were co-localized with CTCF (Fig. S8A and S8B). In contrast, BRG1 and SMARCA5 binding remained largely unaffected (Fig. S8C and S8D). Further analysis revealed that both CHD4 and CHD8 binding were significantly diminished at genomic loci showing accessibility reduction post-CTCF degradation (Fig. S8E). These findings suggest that CTCF enhances chromatin accessibility primarily by stabilizing the binding of CHD4/8 complexes.

Then we knocked either *Chd4* or *Chd8* down to establish functional relationships (Fig. S8F). Loss of CHD4 significantly altered chromatin accessibility, whereas loss of CHD8 had a minor effect, but both losses predominantly led to decreased chromatin accessibility at their binding sites (Fig. S8G and S8H). Moreover, we found that ATAC signals with CTCF binding were significantly downregulated in the promoters of downregulated genes upon CTCF depletion. CHD4 binding decreased at a subset of these gene promoters, while CHD8 binding was reduced at nearly all promoters of downregulated genes (Fig. S8I).

To further correlate the observed reduction in CHD4/8 binding and chromatin accessibility to gene expression changes, we analyzed the expression of genes exhibiting decreased chromatin accessibility at their promoters upon either *Chd4* or *Chd8* knockdown. Given that *Chd8* is expressed at low levels in mESCs and its knockdown resulted in a reduction of chromatin accessibility at the promoters of only two genes, we performed RT-qPCR and found that the expression of both genes was significantly downregulated (Fig. S8J), indicating a functional link between *Chd8* knockdown-induced loss of chromatin accessibility and downregulation of gene expression. *Chd4* is highly expressed in mESCs, and its depletion led to widespread chromatin accessibility changes. We performed RNA-seq and found that *Chd4* knockdown resulted in ­significant transcriptomic alterations compared to the control (Fig. S8K). Integration of ATAC-seq and RNA-seq data revealed that the genes with reduced promoter accessibility were predominantly downregulated (Fig. S8L). Further integration of ATAC-seq and RNA-seq data with CTCF target genes showed that most promoters of CTCF target genes exhibited reduced chromatin accessibility upon *Chd4* knockdown, and nearly half of these genes were also downregulated at the expression level (Fig. S8M), supporting that *Chd4* knockdown-induced loss of chromatin accessibility reduced gene expression. Together, these findings suggest that CTCF facilitates gene expression by enhancing chromatin accessibility through the stabilization of binding of both CHD4 and CHD8, revealing a novel mechanism of CTCF-mediated transcriptional regulation independent of chromatin looping.

CTCF has long been recognized as a key regulator of EP interactions through chromatin loop formation, but acute depletion studies reveal that only a subset of genes and EP interactions are CTCF-dependent. This selectivity suggests that specific genomic or cellular contexts may be required for CTCF-mediated looping to functionally regulate EP interactions. To systematically investigate the determinants of CTCF loop-dependent gene regulation, we performed high-resolution chromatin interaction mapping using MNase-based HiChIP with CTCF and H3K27ac antibodies. Our optimized HiChIP protocol yielded highly reproducible datasets with robust antibody enrichment (Fig. S9A and S9B). Notably, even at low sequencing depth, we were able to clearly capture EP interactions at classical gene loci such as *Sox2* and *Nanog* (Fig. S9C and S9D). ­Compared to Micro-C data with ultradeep sequencing, our HiChIP datasets with approximately 10-fold less sequencing depth still robustly detect both CTCF and H3K27ac-­mediated chromatin interactions at the *Sox2* locus with 1 kb resolution, where the interactions identified in the Micro-C data appeared much weaker (Fig. S9E).

Following stringent filtering to remove short-range interactions, low-count loops, and merging loops from the identical genomic elements, we identified 11,777 high-­confidence H3K27ac-mediated chromatin interactions (referred to as H3K27ac interactions). Classification by anchor types (CTCF, C; enhancer, E; promoter, P; CTCF/enhancer, CE; CTCF/promoter, CP) showed these interactions primarily comprised three functional classes: PP, EE, and EP interactions (Fig. S9F). Genomic annotation of these interaction anchors identified 4,245 distinct promoters and 4,141 enhancers (Fig. S9G). While H3K27ac marks were broadly distributed across the genome (Fig. S9H), only 40.5% (12,006/29,656) of these modified loci participated in significant chromatin interactions (Fig. S9I). Comparative genomic coverage analysis showed that whereas CTCF-mediated loops spaned approximately 51% of the genome, H3K27ac-dependent interactions occupied a much smaller fraction (8.8%) (Fig. S9J and S9H).

Interestingly, H3K27ac-marked regions that failed to mediate chromatin interactions (designated as non-­looping sites) exhibited significantly lower H3K27ac ChIP-seq signals compared to looping sites (Fig. S10A). However, even strong enhancer elements, including the SEs associated with *Ppa2* and *Zbtb39* loci, which showed robust H3K27ac enrichment, failed to establish significant chromatin contacts with their promoters (Fig. S10B and S10C). Furthermore, genomic spatial analysis revealed an additional distinguishing feature: non-looping H3K27ac sites were located substantially farther from neighboring H3K27ac sites compared to looping H3K27ac regions (Fig. S10D).

Acute CTCF degradation had minimal impact on the enrichment of H3K27ac (Fig. S10E) at these sites and on the contact frequency of H3K27ac-mediated interactions (Fig. S10F). Differential analysis revealed that CTCF degradation significantly affected less than 2% (194/11,777, 1.65%) of H3K27ac interactions (Fig. S10G), indicating that H3K27ac interactions are largely insensitive to acute CTCF depletion.

To elucidate the limited impact of CTCF degradation on H3K27ac-mediated interactions, we systematically categorized H3K27ac interactions based on their spatial relationship with CTCF loops into five distinct classes: “contain,” “cross,” “neighbor,” “inside,” and “outside” (Fig. 2A). Given the frequent co-occurrence of multiple CTCF loops near H3K27ac interaction sites, these spatial relationships often exhibited complex configurations. In this study, we specifically focused on the “contain & cross” condition (Fig. 2A).

**Figure 2.**
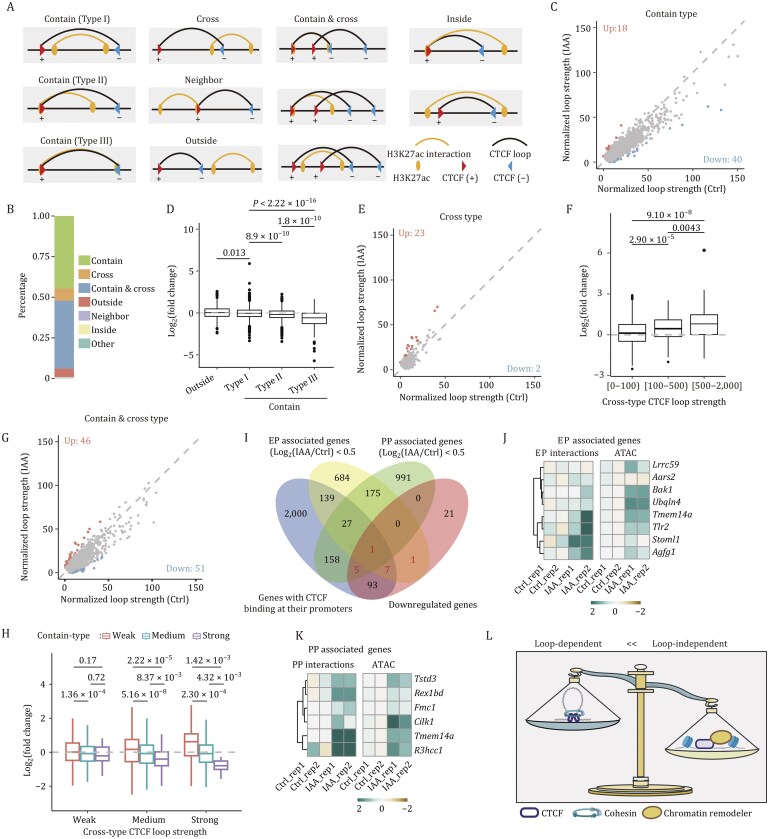
**CTCF predominantly regulates transcription through loop-independent mechanisms**. (A) Schematic diagrams illustrating the classification of H3K27ac interactions based on the positional relationships with CTCF loops. (B) Percentage distribution of different kinds of H3K27ac interaction classified in panel (A). (C) Scatter plot showing the strength of contain-type H3K27ac interactions in untreated and IAA-treated CTCF^AID^ mESCs. (D) Box plots showing the fold change distributions of different types of H3K27ac interaction after CTCF degradation. (E) Scatter plot showing the strength of cross-type H3K27ac interactions in untreated and IAA-treated CTCF^AID^ mESCs. (F) Fold change distribution of cross-type H3K27ac interactions grouped by different strength of overlapped CTCF loops. (G) Scatter plot showing the strength of contain & cross-type H3K27ac interactions in untreated and IAA-treated CTCF^AID^ mESCs. (H) Fold change distribution of contain & cross-type H3K27ac interactions classified by contain-type and cross-type CTCF loop strength. (I) Venn diagram showing overlap among downregulated genes, genes with CTCF binding at their promoters, and genes associated with downregulated EP or PP interactions. Downregulated genes associated with altered EP or PP interactions are highlighted in red. (J) Heatmaps showing the change of EP interactions and ATAC signal at promoters of genes (from panel I) that exhibit downregulated EP interactions and CTCF binding in their promoters. (K) Heatmaps showing the change of PP interactions and ATAC signal at promoters of genes (from panel I) that have downregulated PP interactions and CTCF binding in their promoters. (L) Schematic summary of CTCF loop-dependent and loop-independent functions. In boxplots (D, F, H), the center line indicates the median, the box represents the data between the first and third quantiles, the whiskers indicate the 1.5 interquartile range, and outliers are omitted. *P*-values are calculated by a two-sided Mann–Whitney *U* test.

Our classification revealed that the majority of H3K27ac interactions (nearly 80%) fall into the “contain” and “contain & cross” types, while the “cross” and “outside” types account for less than 10% (Fig. 2B). Detailed examination revealed that CTCF depletion significantly attenuated the interaction strength for the majority of contain-type H3K27ac interactions (Fig. 2C). This effect was particularly pronounced at the *Deptor* and *Dusp27* gene loci (Fig. S11A and S11B), where we observed near-complete loss of interactions following CTCF degradation (Fig. S11C and S11D). These H3K27ac interactions are identical to CTCF loops, with CTCF binding at both anchors, indicating that CTCF directly enhances H3K27ac interactions via chromatin looping.

Subclassification of contain-type H3K27ac interactions uncovered differential sensitivity to CTCF depletion. ­Compared with outside-type interactions, contain-type-I interactions showed minimal response to CTCF degradation. In contrast, both contain-type-II and contain-type-III interactions exhibited significant weakening following CTCF loss, with the latter demonstrating particularly pronounced reduction (Fig. 2D). Although CTCF-mediated looping provides a clear mechanistic basis for the stabilization of contain-type-III interactions, the underlying principles governing CTCF-dependent enhancement of contain-type-II H3K27ac interactions remain to be elucidated.

To investigate the mechanism, we examined the H3K27ac-solo anchors—those enriched for H3K27ac but lacking CTCF binding—from contain-type-II interactions. Genomic segmentation analysis based on histone modifications and CTCF binding sites (Fig. S11E) demonstrated that these H3K27ac-solo anchors were specifically localized to promoter and enhancer regions (Fig. S11F). Intriguingly, high-resolution CTCF MNase HiChIP analysis revealed the formation of stripe-shaped interaction patterns (Fig. S12A), characterized by extensive contacts between CTCF-bound sites and distal regions lacking CTCF occupancy (Fig. S12B). Annotation of these interaction targets showed predominant localization to promoters and enhancers (Fig. S12C), suggesting that CTCF may facilitate chromatin contacts between its binding sites and these regulatory elements through a stripe-mediated mechanism.

We postulated that the cohesin complex may play a key role in mediating these interactions through loop extrusion mechanism. In this model, cohesin may stall at one CTCF-bound site while continuing to extrude DNA from the other direction, thereby establishing relatively stable chromatin contacts either between convergent CTCF sites or between CTCF sites and regulatory elements (promoters/enhancers) (Fig. S12D). To validate this hypothesis, we conducted RAD21 MNase HiChIP experiments, which demonstrated significant RAD21 enrichment at both promoter and enhancer regions, in addition to its expected colocalization with CTCF sites (Fig. S12E–G). Our findings corroborate previous reports that promoters can function as loop extrusion barriers (Busslinger et al., 2017). These results support a model wherein CTCF facilitates H3K27ac interactions by binding to one or both anchors of interaction and coordinates with cohesin to promote same-­directional loops.

Compared to contain-type H3K27ac interactions, cross-type H3K27ac interactions are less frequent (Fig. 2B). Upon CTCF degradation, most cross-type interactions remained largely unaffected, with only a small subset showing notable enhancement (Fig. 2E). A representative example was observed at the *Dppa3* locus, where multiple CTCF loops insulate the promoter from its distal enhancers (Fig. S13A). Upon CTCF degradation, we detected significant chromatin contacts between these regulatory elements, concomitant with substantial upregulation of *Dppa3* expression (Fig. S13B and S13C). Our statistical analysis revealed that weak cross-type CTCF loops had minimal impact on H3K27ac interactions, while stronger CTCF loops with insulating functions exhibited a greater inhibitory effect (Fig. 2F). These findings align with previous reports that strong CTCF loops, mediated by robust or multiple CTCF binding sites, provide substantial insulation (Chakraborty et al., 2023; Chang et al., 2023; Huang et al., 2021; Zuin et al., 2022).

In contrast to “cross-type” interactions, “contain & cross-type” H3K27ac interactions are more abundant (Fig. 2B) and were largely unaffected by CTCF degradation (Fig. 2G). A representative case was observed at the *Ino80*-*Exd1* loci, where multiple CTCF loops simultaneously exerted both promotional and insulating effects on the interacting promoters (Fig. S13D). Following CTCF degradation, we detected strengthened promoter–promoter (PP) interactions (Fig. S13D and S13E), demonstrating that the insulating capacity of CTCF loops predominates over their promotional function in this context. The complex interplay between opposing CTCF activities at shared genomic loci presents unique challenges for studying “contain & cross”-type interactions. Our systematic analysis revealed that the strength of CTCF loops determines its functional outcome. When both CTCF loops (promotional and insulating) were weak, H3K27ac interactions showed minimal response to CTCF depletion (Fig. 2H). Robust CTCF loops—whether promotional or insulating—exerted significant effects on H3K27ac interactions. When the promotional and insulating effects were balanced, their influence was neutral (Fig. S13F); however, when either function predominated, H3K27ac interactions were either enhanced (promotional dominance) or insulated (inhibitory dominance), respectively (Fig. 2H).

To investigate potential functional correlation between altered H3K27ac interactions and DEGs following CTCF degradation, we found that genes linked to downregulated EP interactions showed no significant expression changes, while those associated with upregulated EP interactions exhibited only minor transcriptional increases (Fig. S14A). These observations suggest that EP interactions may be dispensable for expression of these genes. To test this hypothesis, we employed CRISPR/Cas9-mediated deletion of specific enhancers (*Sema4b* and *Chd2* loci) that showed altered EP interactions but unaffected gene expression upon CTCF depletion (Fig. S14B–D). Consistent with our hypothesis, enhancer knockout confirmed that neither these regulatory elements nor their cognate EP interactions were required for target gene expression (Fig. S14E and S14F).

Beyond EP interactions, our analysis revealed extensive PP interactions. However, altered PP interactions generally showed even weaker correlation with gene expression changes than that of altered EP interactions (Fig. S14A and S15A). While most PP interaction modifications had minimal transcriptional consequences, we identified specific loci where coordinated expression changes accompanied PP interaction alterations. For example, interactions between the *Rnf146* and *Echdc1* promoters, as well as between *H2-M5* and *Zfp57* promoters, showed slight upregulation, which was accompanied by increased gene expression (Fig. S15B–E). To further investigate the regulatory role of these PP interactions, CRISPR/Cas9-mediated knockout of either *Rnf146* or *H2-M5* promoters (Fig. S15F and S15G) led to significant downregulation of both interacting gene pairs (Fig. S15H and S15I), demonstrating functional interdependence between these promoters.

Integrative analysis of DEGs revealed that most transcriptional changes occurred independently of altered EP or PP interactions (Fig. S16A), indicating that changes in these interactions were not the primary drivers of differential gene expression. Our findings revealed that CTCF regulates gene expression through both loop-dependent and loop-independent mechanisms (Figs. 1 and 2). To compare these two functions, we performed an integrative analysis of genes with CTCF binding at their promoters, CTCF-regulated genes (Fig. 1H), and genes associated with EP and PP interactions. We found that 72.6% (93/128) of downregulated genes had CTCF binding at their promoters without concomitant reduction in EP or PP interactions (Fig. 2I), suggesting that these genes were regulated through CTCF’s loop-independent function. Merely 15 downregulated genes exhibited diminished EP or PP interactions, with 14 of these having CTCF binding at their promoters (Fig. 2J and 2K), indicative of cooperative regulation by both loop-dependent and loop-independent mechanisms. Collectively, these data establish that CTCF-mediated transcriptional repression operates principally through loop-independent pathways.

Among upregulated genes, only four exhibited enhanced EP or PP interactions (Fig. S16B and S16C), including *Dppa3* (Fig. S13A–C). Interestingly, the majority of upregulated genes displayed comparable rescue patterns upon ­reintroduction of either CTCF^wt^ or CTCF^mut^ (Fig. S16D). This observation strongly supports the conclusion that CTCF-mediated upregulation occurs predominantly through loop-independent mechanisms.

In conclusion, our findings show that in the context of acute CTCF degradation, the loop-independent functions of CTCF play a more significant role in gene regulation than its loop-dependent functions (Fig. 2L).

## Supplementary data

Supplementary data is available at *Protein & Cell* online https://doi.org/10.1093/procel/pwae087.

## Footnotes

This work was supported by the National Natural Science Foundation of China (32430016 and U21A20195), the National Key R&D Program of China (2021YFA1100300), the Guangzhou Municipal Science and Technology Program (2023B03J1230), and the Major Project of Guangzhou National Laboratory (GZNL2023A02010 and GZNL2023A02008). The authors gratefully thank the support from the Guangzhou Branch of the Supercomputing Center of the Chinese Academy of Sciences.

The authors gratefully thank the support from the Guangzhou Branch of the Supercomputing Center of the Chinese Academy of Sciences. The authors declare no competing interests. All authors give their consent to ­participate and for publication.

H.Y. and G.H. initiated the study and designed the experiments. G.H. performed the bioinformatics analysis. B.J. conducted most of the experiments. J.Z. conducted BL-Hi-C and ATAC-seq experiments. B.J. and J.Z. conducted QHR-4C and M4C experiments. Y.Lu conducted TT-seq experiments, X.W. conducted Co-IP experiments. B.J. and Y.Liu conducted BIOTIN ChIP-seq experiments. R.W. and J.L. provided necessary assistance. H.Y., G.H., and J.Z. wrote the manuscript. H.Y. conceived and supervised the entire study. No AI tools were used in this manuscript.

The RNA-seq, TT-seq, ChIP-seq, ATAC-seq, M4C, BL-HiC, and MNase HiChIP data reported in this paper have been deposited in the Gene Expression Omnibus (GEO) database under accession number GSE275003 and Genome Sequence Archive (GSA) database under accession number CRA027761. Published RNA-seq data (GSE146454, GSE178982 and GSE98671), ChIP-seq data in 2i-cultured mESCs (H3K4me3, GSE186349; H3K4me1, GSE72164; H3K27ac, GSE186349; H2BK20ac, GSE186349; MED1, GSE186349; RING1B, GSE72164; SUZ12, GSE23943) and Micro-C data (GSE130275) were used in this paper. The code for analyzing M4C was included in the github (github.com/hugch2020/M4C).

## Supplementary Material

pwaf087_Supplementary_Data
