# Supplementary material for: CTCF’s loop-independent functions prevail over chromatin looping in the acute degradation system
Source: Protein Cell. 2025 Nov 4;17(4):357–65. doi: 10.1093/procel/pwaf087 (PMC13107559; doi:10.1093/procel/pwaf087)
Supplement: pwaf087_Supplementary_Data [file pwaf087_supplementary_data.pdf]

I: Materials and Methods

II: Supplementary Figures and Figure legends

III: Supplementary Methods

IV: Supplementary Tables

I: Materials and Methods

Cell culture

HEK293T cells were cultured in DMEM high-glucose media (Hyclone, Cat# SH30022.01) supplemented with 10% FBS (Natocor, Cat# SFBE) under 5% CO_2 at 37°C. Mouse CTCF-AID ESCs (a kind gift from Benoit Bruneau) and RAD21 AID cells were cultured on 0.2% gelatin-coated dishes in serum-free medium under 5% CO_2 at 37°C. The serum-free medium includes 48% DMEM/F12 (Thermo Fisher Scientific, Cat# 11330-032), 48% Neurobasal (Thermo Fisher Scientific, Cat# 21103-049), 0.5% N2 (Thermo Fisher Scientific, Cat# 17502-048), 1% B27 (Thermo Fisher Scientific, Cat# 12587-010), 1000 U/mL leukemia inhibitory factor (LIF) (Millipore, Cat# ESG1107), 1 μM PD0325901 (Selleck, Cat# S1036), 3 μM CHIR99021 (Selleck, Cat# S2924), 1 mM non-essential amino acid (Gibco, Cat# 11140050), 1 mM GlutaMAX (Gibco, Cat# 35050061) and 0.1 mM β-mercaptoethanol (Life Technologies, Cat# 21985023). CTCF degradation was induced by treating cells with a final concentration of 500 μM indole-3-acetic acid (IAA, Solarbio, Cat# I8780). RAD21 degradation was induced by treating cells with a final concentration of 1 μM 5-Ph-IAA (BioAcademia, Japan, Cat# 30-003).

Plasmid construction

For the generation of *Ctcf* overexpression vectors, the *Ctcf* cDNAs were amplified from the cDNA of mESCs and then cloned into the pSIN-Flag-HA vector. Y226A/F228A double mutations were introduced with site-directed

mutagenesis and inserted into the pSIN-Flag-CTCF-HA vector. For *Chd4* or *Chd8* knockdown, the shRNA oligos targeting either *Chd4* or *Chd8* were inserted into the lentiviral vector pLKO.1. The sequences of shRNA oligos are listed in Table S1.

For generating CTCF motif, enhancer or promoter deleted clones by using CRISPR/Cas9, specific sgRNA oligos were synthesized, annealed and then cloned into the pX459 vector (pSpCas9(BB)-2A-Puro, Addgene, Cat# 62988). The sequences of oligos are listed in Table S2. For the generation of in vivo biotin-tagged *Chd4*, *Chd8*, *Smarca4* or *Smarca5* mESCs, two homology arms (~1kb each) around the stop codon of the indicated gene were amplified by PCR from genomic DNA of mESCs. Then, the homology arms and FLAG-Avitag-mCherry tag were cloned into pMD18-T plasmid (Takara, Cat# 6011). sgRNAs targeting on *Chd4/Chd8/Smarca4/Smarca5* were synthesized, annealed and then cloned into the pX459 vector, respectively. The sequences of oligonucleotides are listed in Table S2.

Generation of *Rad21*-auxin-induced degron (*Rad21*-AID) mESCs

For the generation of RAD21 auxin-induced degron (Rad21-AID) mESCs, two homology arms (~1kb each) around stop codon of the gene were amplified by PCR from genomic DNA of E14 mESCs. Then, the homology arms and miniAID-eGFP tag were cloned into pMD18-T plasmid as donor vector. Rad21-targeting sgRNAs were synthesized, annealed and then cloned into the pX330 vector (Addgene, Cat# 42230). The sequences of oligonucleotides are listed in Table S2.

E14 mESCs were infected with *OsTir1(F74G)*-V5 lentivirus and selected with 10 μg/mL blasticidin (Yeasen, Cat# 60218ES10) for 5 days. *OsTir1(F74G)*-V5 overexpression was verified with anti-V5 antibody by Western blot. The *OsTir1(F74G)*-V5-overexpressing mESCs were transfected with 1 μg of donor vector and 1 μg of Cas9-sgRNA vector using FuGENE HD transfection reagent. After transfection, eGFP-positive cells were sorted into a

48-well plate for single-clone selection. Single clones were genotyped by PCR for homozygous insertion of miniAID–eGFP and further validated by Sanger sequencing.

Generation of *Ctcf*^{wt}/*Ctcf*^{mut} stably overexpressing mESCs or *Chd4/Chd8*-depleted mESCs

Lentivirus for pSin or pLKO.1 was assembled with the packaging plasmids (psPAX2 and pMD2.G) in HEK293T cells. Viral supernatants were collected at 24 h and 48 h post-transfection, respectively, filtered and then mixed with 8 μg/mL polybrene to infect mESCs. Stable cell lines were selected with 2 μg/mL puromycin 24 h after infection.

Generation of mESCs with the deletion of CTCF motif, enhancer and promoter, respectively

The CRISPR/Cas9 system was used to construct the genome-edited cell lines. SgRNAs were designed by an online website tool (http://benchling.com). The primers were synthesized, annealed and then cloned into the the pX459 vector. 5 x 10^4 mESCs were transfected with 2 μg of Cas9-sgRNA vector using FuGENE HD transfection reagent (Promega, Cat# E2311). Cells were selected with 2 μg/mL of puromycin (Gibco, Cat# A11138-03) after 24 h of infection and selected for 48 h. Single clones were picked and transferred into 48-well plates for subsequent identification by PCR and Sanger sequencing to obtain correctly edited clones.

Generation of *in vivo* biotinylated mESCs

The CTCF-AID mESCs were infected with lenti-birA-V5 lentivirus and selected with 10 μg/mL blasticidin (Yeasen, Cat# 60218ES10) for 5 days. BirA-V5 overexpression was confirmed with anti-V5 antibody by Western blot. Then BirA-V5-overexpressing cells were transfected with 1 μg of donor and 1 μg of Cas9-sgRNA using FuGENE HD transfection reagent. Cells were selected with

2 μg/mL of puromycin for 48 h. Single clones were picked and transferred to 48-well plates for subsequent identification by PCR and Sanger sequencing to obtain correctly edited clones. Finally, *in vivo* biotinylation for CHD4, CHD8, SMARCA4 or SMARCA5 was detected with anti-Streptavidin (HRP) antibody.

Chromatin immunoprecipitation with reference exogenous genome (ChIP-Rx) and biotin chromatin immunoprecipitation (Biotin ChIP)

For CTCF, SMC1 ChIP-Rx, mES cells were crosslinked with 1% formaldehyde for 10 min at room temperature (RT). For BIOTIN ChIP for the remodeler factors (CHD4, CHD8, SMARCA4, and SMARCA5), mES cells were crosslinked with 2 mM DSG (Disuccinimidyl glutarate, Thermo Fisher scientific, Cat# 20593) for 30 min and 1% formaldehyde for 10 min with rotation at RT. The reaction was stopped by adding glycine to a final concentration of 125 mM. Crosslinked cells were lysed in ChIP SDS lysis buffer (1% SDS, 10 mM EDTA, 50 mM Tris-HCl (pH 8.0)) containing 1x protease inhibitor cocktail and PMSF, then sonicated to achieve a chromatin sized of 200–400 bp (Bioruptor Plus sonicator). The supernatant was collected through centrifugation at 14000 x g for 15 min. For CTCF, SMC1A ChIP-Rx, the supernatant was mixed with 10% of lysate supernatant of HEK293T cells, which was processed same as mESCs, as the spike-in for normalization. 1% of the total supernatant for input were collected, and the remaining supernatant were diluted 9-fold with dilution buffer (0.01% SDS, 1.1% Triton X-100, 1.2 mM EDTA, 16.7 mM Tris-HCl (pH 8.0) and 167 mM NaCl), precleared with Dynabeads protein A/G (1:1 mixed) at 4°C for 1 h and immunoprecipitated using 4 μg antibodies for 12 h and 50 μl Dynabeads protein A/G (1:1 mixed) for 3 h. Immune complexes were washed with the following buffers: low salt wash buffer (0.1% SDS, 1% Triton X-100, 2 mM EDTA, 20 mM Tris–HCl (pH 8.0) and 150 mM NaCl), high salt wash buffer (0.1% SDS, 1% Triton X-100, 2 mM EDTA, 20 mM Tris–HCl (pH 8.0) and 500 mM NaCl), LiCl wash buffer (0.25 M LiCl, 1% IGEPAL-CA630, 1% deoxycholic acid (sodium salt), 1 mM EDTA and 10 mM Tris–HCl (pH 8.0)) and TE buffer

(10 mM Tris–HCl (pH 8.0) and 1 mM EDTA). ChIPed DNA was reverse-crosslinked and purified for the qPCR analysis or library generation. ChIP-seq libraries were constructed using the VAHTS Universal DNA Library Prep Kit for Illumina®V3 (Vazyme Biotech, Cat# ND607) according to the manufacturer's instructions. The primers used in the ChIP-qPCR assays are listed in Table S3.

Western blot

Cells were collected and lysed in RIPA buffer (0.1% SDS, 1% Triton X-100, 150 mM KCl, 50 mM Tris-HCl (pH 7.4), 1 mM EDTA, 1 mM PMSF and 1× protease inhibitor cocktails) on ice for 30 min. Total soluble proteins were obtained by centrifugation at 12000 rpm for 10 min. After centrifugation, the samples were separated on SDS-PAGE gel and transferred to polyvinylidene fluoride (PVDF) membranes (Millipore, Cat# IPVH00010). The PVDF membrane was blocked with 5% milk in TBS-T (TBS with 0.05% Tween-20) and incubated with corresponding primary antibody and secondary antibody. The antibodies used in this study are listed in Table S4.

Co-immunoprecipitation (co-IP)

Nuclear protein extracts were prepared from mESCs. 1 mg of proteins were used for each co-IP experiment. The protein samples were incubated with 2 μg of antibodies overnight. Following the incubation, 15 μL of protein A (Invitrogen, Cat# 10001D) and 15 μL of protein G (Invitrogen, Cat# 10004D) dynabeads were added to the samples and incubated at 4°C for 3 h. After incubation, the beads were washed three times with IP wash buffer (50 mM Tris-HCl (pH7.6), 150 mM KCl, 0.1% Triton X-100, 1 mM EDTA and 1 × protease inhibitor cocktail). The bound proteins were then eluted from the beads, loaded onto SDS-PAGE gel and transferred to PVDF membrane. Then the membrane was incubated with the indicated antibodies. Antibodies are detailed in Table S4.

RT-qPCR

Total RNAs were extracted from cells with a RaPure Total RNA Micro kit (Magen, Cat# R4012-03). RNA quantification and reverse transcription were performed by HiScript® III RT SuperMix for qPCR (+gDNA wiper) (Vazyme Biotech, Cat# R323-01). Real-time PCR was performed with SYBR Green mix (Genstar, Cat# A301-01) on a CFX96 real-time PCR system (Bio-Rad) according to the manufacturer's instructions. The primers used in the RT-qPCR assays are listed in Table S5.

RNA sequencing

Total RNAs were extracted as described above. RNA sequencing libraries were constructed using the VAHTS Universal V8 RNA-seq Library Prep Kit for Illumina (Vazyme Biotech, Cat# NR605). Two rounds of mRNA purification were performed to guarantee the removal of rRNA. The mRNAs were fragmented to 250-450 bp at 85°C for 6 min, followed by immediate cDNA first- and second-strand synthesis, end-repair and adapter ligation reactions. cDNA purification and size selection were performed by AMPure XP beads (Beckman Coulter, Cat# A63882). The product was used for library amplification and purification followed by sequencing on an Illumina NovaSeq platform (Annoroad Gene Technology Co., Ltd.).

RNA-seq analysis

Raw reads were subjected to TrimGalore (version 0.6.5, https://www.bioinformatics.babraham.ac.uk/projects/trim_galore/) to remove adaptors and low quality reads. Trimmed reads were first aligned to rRNA sequences to remove rRNA by bowtie2 (version 2.2.5) (Langmead and Salzberg, 2012), unmapped reads were then subjected to STAR-RSEM pipeline mapping to mm10 genome. RSEM (version 1.2.22) (Li and Dewey, 2011) was used for transcript quantification. DESeq2 (version 1.32.0) (Love et al., 2014) was used to obtain normalized counts and identify differentially

expressed genes, which were at least 1.5-fold up- or down-regulated, with q-values less than 0.05. Lowly expressed genes with TPM (transcripts per kilobase of exon model per million mapped reads) value less than 1 were filtered out.

ChIP-seq analysis

Adaptor sequences from raw reads were trimmed using TrimGalore with default options. For CTCF and SMC1A ChIP-Rx data, trimmed reads were mapped to the mouse (mm10) and human (hg38) mixed genomes using bowtie2 with the following parameters: --very-sensitive --end-to-end --no-unal --no-mixed --no-discordant. Aligned reads showing PCR duplication, having mapping quality lower than 30, or overlapping with Encode blacklist regions were removed. Reads mapped to the mouse or human genome were separated and then subjected to peak calling using macs2 (version 2.2.7.1) (Zhang et al., 2008) with the default parameters. Scale factors were calculated as described in previously published paper (Zhang et al., 2024). Normalized bigwig files were generated with the bamCoverage tool in deepTools2 (version 3.5.1) (Ramirez et al., 2016) using RPGC normalization and scale factors.

For BIOTIN ChIP-seq data, trimmed reads were mapped to mm10 genome using bowtie2 with parameters "--very-sensitive --end-to-end --no-unal --phred33 -X 2000 --no-mixed --no-discordant". Only uniquely mapped reads with MAPQ > 30 were retained. Reads overlapped with Encode blacklist regions of the genome were removed. The reads of few samples with high sequencing reads were subsampled to similar sequencing level. Peaks were called using MACS2 with default parameters. Differential analysis was performed using Diffbind (version 3.2.4) (Ross-Innes et al., 2012) with DESeq2 algorithm. Normalization factors calculated from Diffbind were extracted and divided by 1, which were used as scale factors for bamCoverage tool to generate normalized bigwig files with RPGC normalization. Statistic data from ChIP-seq processing steps are listed in Table S6 and S7.

CTCF motif was scanned with FIMO tools (version 5.5.3) (Grant et al., 2011) from the MEME suite with the default parameters, CTCF motif with the minimal *p* value in each peak was chosen. CTCF peaks were annotated to mouse mm10 RefSeq genes by using ChIPseeker (v1.28.3) (Yu et al., 2015), genes with CTCF binding at their promoters were defined when CTCF binding within ± 500 bp to transcription start site.

BL-Hi-C

The BL-Hi-C libraries were performed as previous described (Liang et al., 2017). Briefly, 1–2.5 × 10^5 cells were treated with 1% formaldehyde for 10 min at RT, and the crosslink was quenched by adding 2.5 M glycine to a final concentration of 125 mM. Then, the cells were lysed with 0.1% SDS FA lysis buffer and then 1% SDS FA lysis buffer. After that, the genome was digested with HaeIII enzyme into fragments with blunt-ends, treated with adenine and ligated with bridge linker containing biotin at 16°C for 4 h. The unligated linker were digested with DNA exonuclease. Next, the cells were treated with SDS and proteinase K to digest proteins, and the DNA was purified using phenol-chloroform extraction with ethanol precipitation. The DNA fragments were sonicated to 300 bp on average. Biotin-labeled DNA fragments were pulled down with Dynabeads™ M-280 streptavidin (Thermo Fisher Scientific, Cat# 11205D) for DNA library construction followed by sequencing.

BL-Hi-C analysis

Raw sequence data were subjected TrimGalore to remove adaptors, and then subjected to trimLinker tool from ChIA-PET2 (version 0.9.3) (Li et al., 2017) to remove linker sequences with parameters "-m 1 -k 2 -e 1 -L 15 -A ACGCGATATCTTATC -B AGTCAGATAAGATAT". The trimmed reads were processed using HiC-Pro (version 2.11.1) (Servant et al., 2015). Raw contact matrices were subsampled to 40 million reads and then transformed into hic format using juicer tools (version 1.22.01) (Durand et al., 2016) with KR

normalization. Compartments were analyzed using juicer tools with default parameters at 100 kb resolution. APA analysis was performed using juicer tools. The reproducibility of BL-Hi-C datasets was calculated on pairs of Hi-C contact matrices at 100 kb resolution using HiCRep (version 0.2.6) (Yang et al., 2017).

TT-seq

TT-seq was performed following previously described with minor modifications (Hu et al., 2023). Exponentially growing cells in 10 cm dish were labeled with 400 μM 4-thiouridine (4sU) (Sigma, Cat# T4509) at 37°C for 15 min under 5% CO_2. The cells were lysed in 3 mL TRIzol (MRC, Cat# TR118) and total RNA was extracted following TRIzol protocol. 4sU-labeled *Drosophila melanogaster* S2 total RNA as spike-in was mixed with collected RNA. 100 μg mixed RNA was fragmentated using NaOH (0.2 M as a final concentration) for 20 min and neutralized with Tris-HCl (pH 6.8) (0.5 M as a final concentration) immediately followed by RNA purification with isopropanol precipitation. Labelled RNA was biotinylated at RT for 3 h and purified with chloroform extraction. The denatured biotinylated RNA was bound with Dynabeads™ MyOne™ Streptavidin C1 (Invitrogen, Cat# 65001) at RT for 15 min, eluted with 100 mM DTT and subsequently purified with RNA clean beads (Vazyme Biotech, Cat #N412). Strand-specific libraries were generated with the VAHTS Universal V8 RNA-seq Library Prep Kit for Illumina (Vazyme Biotech, Cat# NR605) and sequenced with a DNBSEQ-T7 platform (Geneplus, Beijing, China).

TT-seq analysis

Raw data were first subsampled to 70 million reads, and then adaptor sequences were removed by TrimGalore. Trimmed reads were aligned to mouse mm10 genome or spike-in (*Drosophila melanogaster*) genome by STAR aligner (version 2.7) (Dobin et al., 2013). Low mapping quality reads and PCR duplicates were removed by sambamba. FeatureCounts (version 2.0.0)

(Liao et al., 2014) were used to obtain gene level expression with parameters "-t gene -g gene_id -s 2 -p". Gene-level count matrix from *Drosophila* were passed to the estimateSizeFactors function in the DESeq2 package to obtain scale factors, which were used to normalize mouse gene expression.

ATAC-seq

In brief, 5 ×10^4 cells were harvested and washed once with 50 µL of cold PBS. Then the cells were resuspended in 50 µL lysis buffer (10 mM Tris-HCl (pH 7.4), 10 mM NaCl, 3 mM $MgCl_2$, 0.2% IGEPAL CA-630). Then the suspension of nuclei was centrifuged 500 x g at 4°C for 10 min. The pellet was resuspended by adding 50 µL transposition reaction mix (10 µL TD buffer, 5 µL Tn5 transposase and 35 µL nuclease-free H_2O) (Vazyme Biotech, Cat# TD501), and incubated at 37°C for 30 min. Finally, DNA was isolated using MinElute PCR Purification Kit (QIAGEN, Cat# 28106). ATAC-seq libraries were constructed and purified with AMPure XP beads.

ATAC-seq analysis

Paired-ends reads were trimmed using TrimGalore. Trimmed reads were then aligned to the mouse reference genome (mm10) using bowtie2 with parameters "--very-sensitive --end-to-end --no-unal -X 2000". Low mapping quality (MAPQ <30) and unproperly paired alignments, PCR duplicates and alignments located in Encode blacklisted regions and in chrM were filtered out. For CTCF acute depletion ATAC-seq data, alignments with fragment length less than 100 bp were selected. Filtered alignments were subjected to MACS2 to identify peaks with default parameters. Differential binding sites were identified by DiffBind using DESeq2 algorithm. Binding sites with fold change greater than 2 and FDR less than 0.05 were considered as significantly differential binding sites. For *Chd4* and *Chd8* knockdown ATAC-seq data, sites with fold change larger than 1.5 and FDR less than 0.05 were considered as significantly differential binding sites.

QHR-4C and M4C analysis

QHR-4C was performed and analyzed as previously reported (Jia et al., 2020). Detailed MNase-4C protocol is provided in Supplementary Methods. Raw reads were first trimmed using TrimGalore, either read 1 or read 2 after adaptor removal less than 30 bp were filtered out, and then 5’ of read 1 containing primer sequences were selected using cutadapt (version 3.4) with parameters “-g primer --action none --discard-untrimmed”. Selected reads were mapped to mm10 genome using bwa-pairtools workflow to identify chromatin interaction pairs, duplicates were removed, and only “UU, uu, Uu or uU” pair types were kept.

To further filter out non-specific interactions, 300 bp extended from the primer start point along the primer direction was set as the anchor region. Interaction pairs overlapped with the anchor region were extracted.

The selected reads were extended to 150 bp for CTCF loops and 300 bp for EP interactions or polycomb-mediated chromatin interactions, and then converted into bigwig signal file with parameters “-of bigwig -bs 1 --normalizeUsing RPGC --effectiveGenomeSize 1000000” for visualization. Specific primers used for M4C in this study are listed in Table S8, statistic and qualification of M4C data are listed in Table S9.

MNase HiChIP analysis

Detailed MNase HiChIP protocol is provided in Supplementary Methods. Raw reads were trimmed to remove adaptors with TrimGalore, either read 1 or read 2 after adaptor removal less than 30 bp were filtered out. Trimmed reads were handled with bwa-pairtools workflow to generate pair files. Replicates were merged. Anchor 1 and anchor 2 from pair files were extracted from the pair files and extended to 150 bp, which was subjected to macs2 to identify enriched regions with parameters “-g mm --nomodel --extsize 300 --keep-dup all”. For CTCF MNase HiChIP data, parameters “-q 1e-9” was used. For

RAD21 MNase HiChIP data, parameters “-q 1e-6” was used. Peaks were filtered out those that had no CTCF motif, which was scanned by FIMO with default parameters. For H3K27ac MNase HiChIP data, macs2 parameters “--broad --broad-cutoff 1e-5” was used. Peaks were extended 500 bp in both directions and merged if any peak were overlapped.

The merged pairs were transformed into hic format for visualization using juice tools. BigWig files were generated with bamCoverage from deeptools with RPGC normalization. Correlation was evaluated with HiCRep. 5 kb cool files were subjected to HiCRep with each chromosome without chrM, correlation of each chromosome was averaged. Statistic and quality control information are listed in Table S10, S11, S12.

Loop identification for MNase HiChIP

Chromatin interactions were identified by FitHiChIP tool (Version 11.0) (Bhattacharyya et al., 2019). For CTCF, RAD21 MNase HiChIP data, 1 kb resolution, p2p mode with coverage normalization was used.

For H3K27ac MNase HiChIP, 2 kb resolution, p2a mode with coverage normalization was used. The raw loops were handled with several further steps: (1) Filtering short-distance loops. The identified loops with distance less than 100 kb might have false positives. We further identified chromatin loops with 1 kb resolution. The 2 kb resolution loops that overlapped with 1 kb loops were kept. (2) Filtering weak loops. If the maximum count number for each chromatin loop in control and CTCF depleted samples is less than 10, these loops were deemed weak loops and potential false positives. These weak loops were filtered out. (3) Merging loops mediated by the same chromatin element. Chromatin elements such as promoters or enhancers can span more than 2 kb. Loops mediated by these regions would be separated into several loops at a 2 kb resolution. To accurately reflect the interaction strength mediated by these regions, the separated loops must be merged. To fulfill this, the filtered loops were annotated to CTCF, enhancer and promoter regions,

respectively, with GenomicInteractions package (version 1.26.0) (Harmston et al., 2015). Loops were merged if adjacent loops were annotated with the same anchor.

Differential H3K27ac-mediated chromatin interactions

For H3K27ac MNase HiChIP data, the pairs were transformed into bedpe format, then submitted to multiHiCCompare (version 1.16.0) (Stansfield et al., 2019) to perform loess normalization with parameter A.min=3. Normalized loop counts were merged based on above methods, then differential loops were calculated with edger (version 4.0.16) (Robinson et al., 2010). Loops with fold change larger than 1.5 and FDR less than 0.1 were considered as differential loops.

Genome segmentation

The mouse genome was segmented into 14 different chromatin states using chromHMM (version 1.25) (Ernst and Kellis, 2012) with the binding sites from CTCF and MED1 and the peaks from several histone markers (including H3K4me1, H3K4me3, H3K27ac, H3K27me3, H3K9me3, and the H2BK20ac marker reported to be a better indicator of active enhancers (Narita et al., 2023)).

II: Supplementary Figures and Figure legends

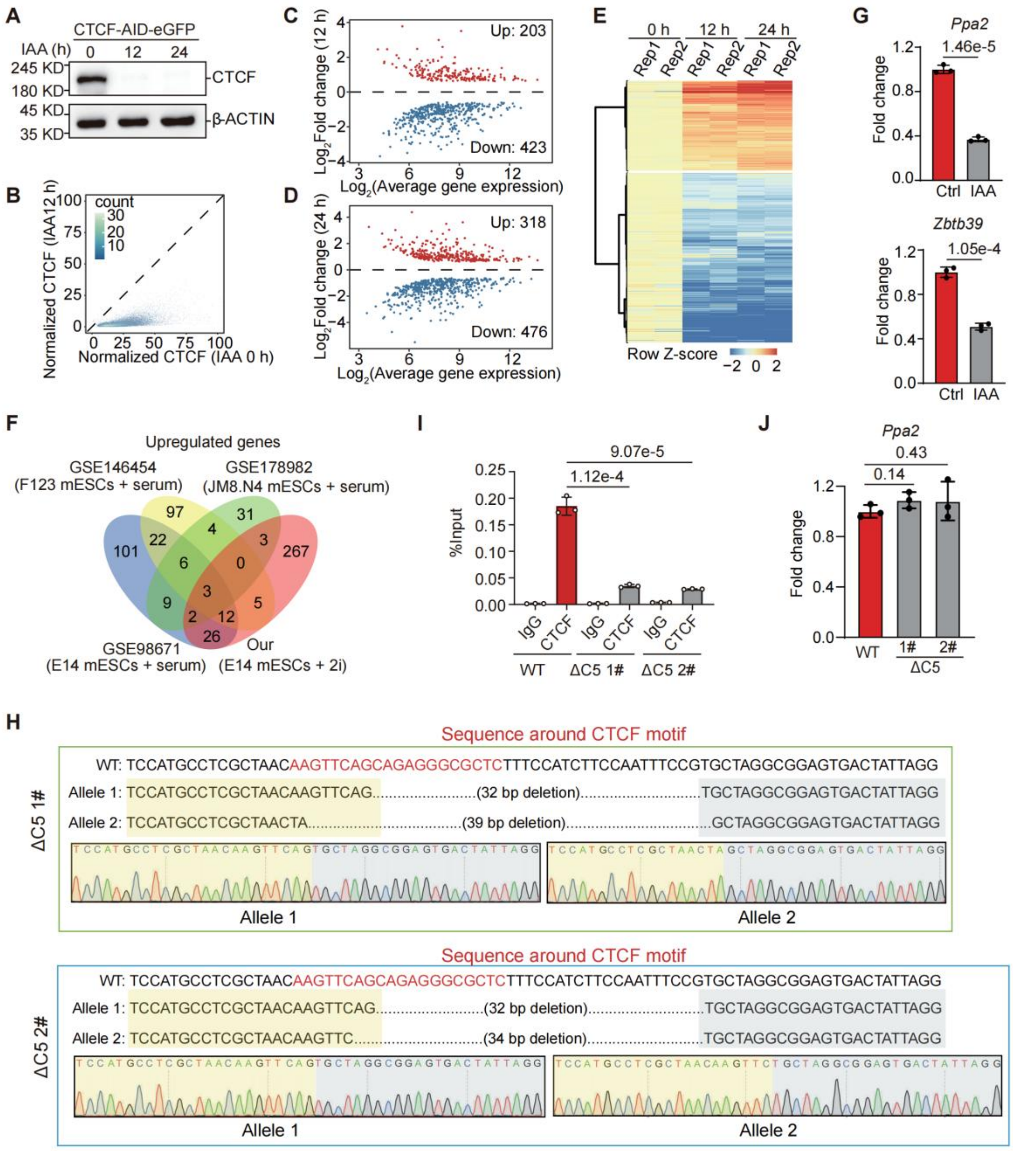

Figure S1. Analysis of CTCF's regulatory role on gene expression following its degradation. (A) Western blot detecting CTCF protein level under 0, 12 and 24 h of IAA treatment in CTCF^{AID} mESCs. (B) Scatter plot showing the changes of CTCF enrichment in CTCF^{AID} mESCs between 0 and 12 h of IAA treatment. (C and D) Scatter plots showing DEGs in CTCF^{AID} mESCs after IAA treatment for 12 h (panel C) or 24 h (panel D). (E) Expression patterns of combined DEGs in CTCF^{AID} mESCs at 12 h or 24 h after IAA

treatment. (F) Overlap analysis of upregulated genes following 24 h of CTCF degradation across our and previously published RNA-seq datasets. (G) RT-qPCR analysis of the expression of *Ppa2* and *Zbtb39* in untreated and 12 h IAA-treated CTCF^{AID} mESCs. (H) Schematic and Sanger sequencing depicting the genomic sequences at the *Ppa2* locus in C5 site-deleted mESCs. (I) ChIP-qPCR analysis of CTCF enrichment at the C5 site in wild-type and CTCF motif-depleted cells. (J) RT-qPCR analysis of *Ppa2* gene expression in wild-type and CTCF motif-depleted cells. In bar plots (G, I, J), quantitative data are presented as mean ± SD and *P* values are calculated by two-sided unpaired *t*-test (n=3 independent biological replicates).

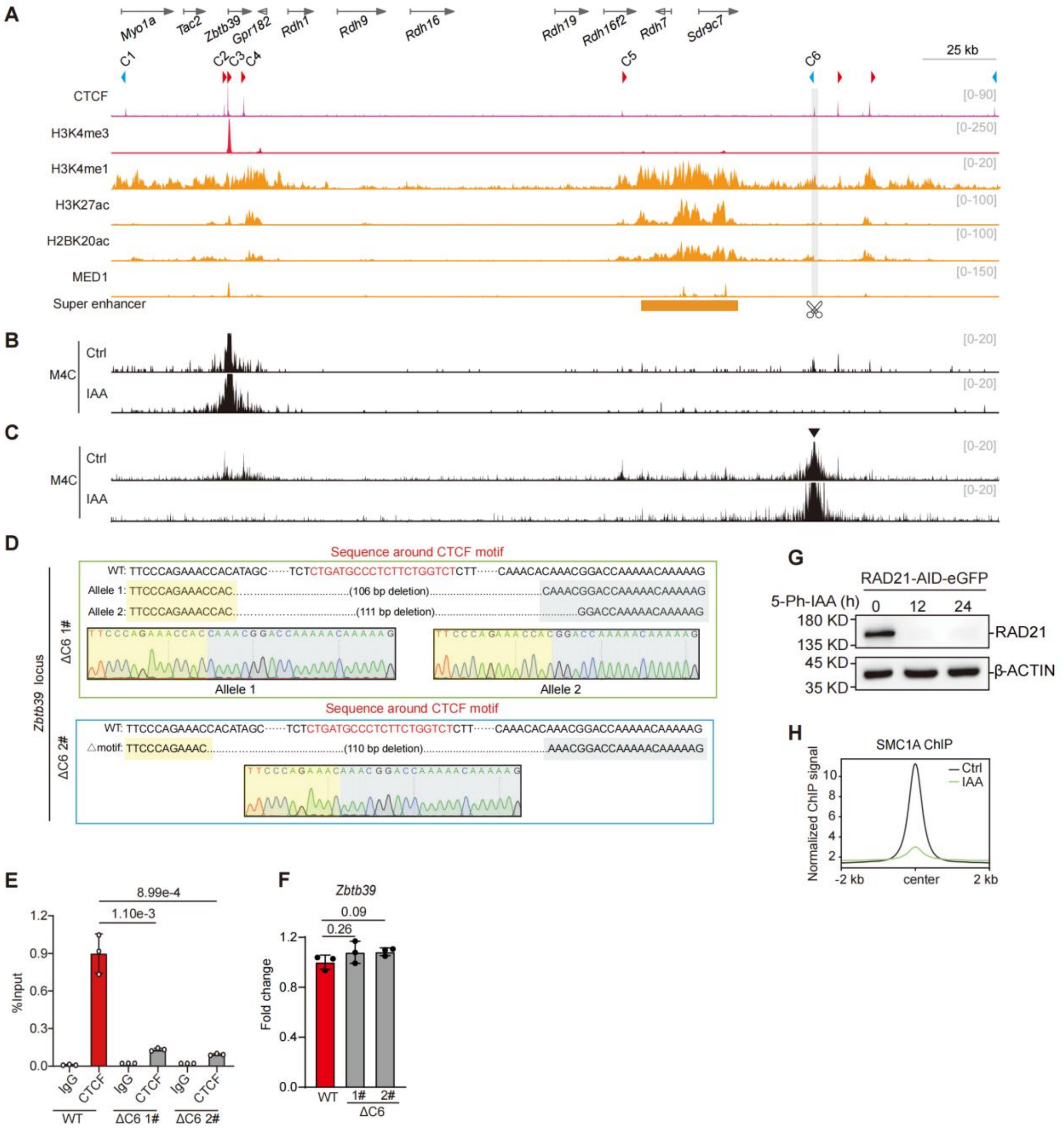
A
Myo1a
Tac2
Zbtb39
Gpr182
Rdh1
Rdh9
Rdh16
Rdh19
Rdh16f2
Rdh7
Sdr9c7
25 kb
C1
C2
C3
C4
C5
C6
CTCF
H3K4me3
H3K4me1
H3K27ac
H2BK20ac
MED1
Super enhancer
[0-90]
[0-250]
[0-20]
[0-100]
[0-100]
[0-150]
B
M4C
Ctrl
IAA
[0-20]
C
D
Sequence around CTCF motif
WT: TTCCCAGAAACCACATAGC······TCTCTGATGCCCTCTTCTGGTCTCTT······CAAACACAAACGGACCAAAAACAAAAAG
Allele 1: TTCCCAGAAACCAC...(106 bp deletion)...CAAACGGACCAAAAACAAAAAG
Allele 2: TTCCCAGAAACCAC...(111 bp deletion)...GGACCAAAAACAAAAAG
Allele 1
Allele 2
ΔC6 1#
Zbtb39 locus
ΔC6 2#
△motif: TTCCCAGAAAC...(110 bp deletion)...AAACGGACCAAAAACAAAAAG
G
RAD21-AID-eGFP
5-Ph-IAA (h)
0
12
24
180 KD
135 KD
45 KD
35 KD
RAD21
β-ACTIN
H
SMC1A ChIP
Normalized ChIP signal
Ctrl
IAA
-2 kb
center
2 kb
E
%Input
8.99e-4
1.10e-3
IgG
CTCF
WT
ΔC6 1#
ΔC6 2#
F
Zbtb39
Fold change
0.09
0.26
WT
1#
2#
ΔC6

Figure S2. CTCF-mediated gene expression regulation through loop-independent mechanisms. (A) Genomic tracks showing the enrichments of CTCF, histone markers, and super enhancer at *Zbtb39* locus (chr10: 127,700,000-128,000,000). The red or blue triangles represent CTCF motifs with forward or reverse direction, respectively. (B) M4C detecting chromatin interactions mediated by *Zbtb39* promoter. (C) M4C detecting chromatin interactions mediated by C6 site at the *Zbtb39* locus. (D) Schematic and Sanger sequencing depicting the genomic sequences at the *Zbtb39* locus in C6 site-deleted mESCs. (E) ChIP-qPCR analysis of CTCF enrichment at the C6 site in control and C6 site-deleted mESCs. (F) RT-qPCR analysis of *Zbtb39* gene expression in control and two C6-deleted mESCs. (G) Western blot analysis of the RAD21 protein level in $RAD21^{AID}$ mESCs following 0, 12 and 24 h of IAA treatment. (H) Average enrichment of SMC1A, a core subunit of cohesin complex, in $RAD21^{AID}$ mESCs following 0 and 12 h of IAA treatment. In bar plots (E, F), quantitative data are presented as mean ± SD and *P* values are calculated by two-sided unpaired *t*-test (n=3 independent biological replicates).

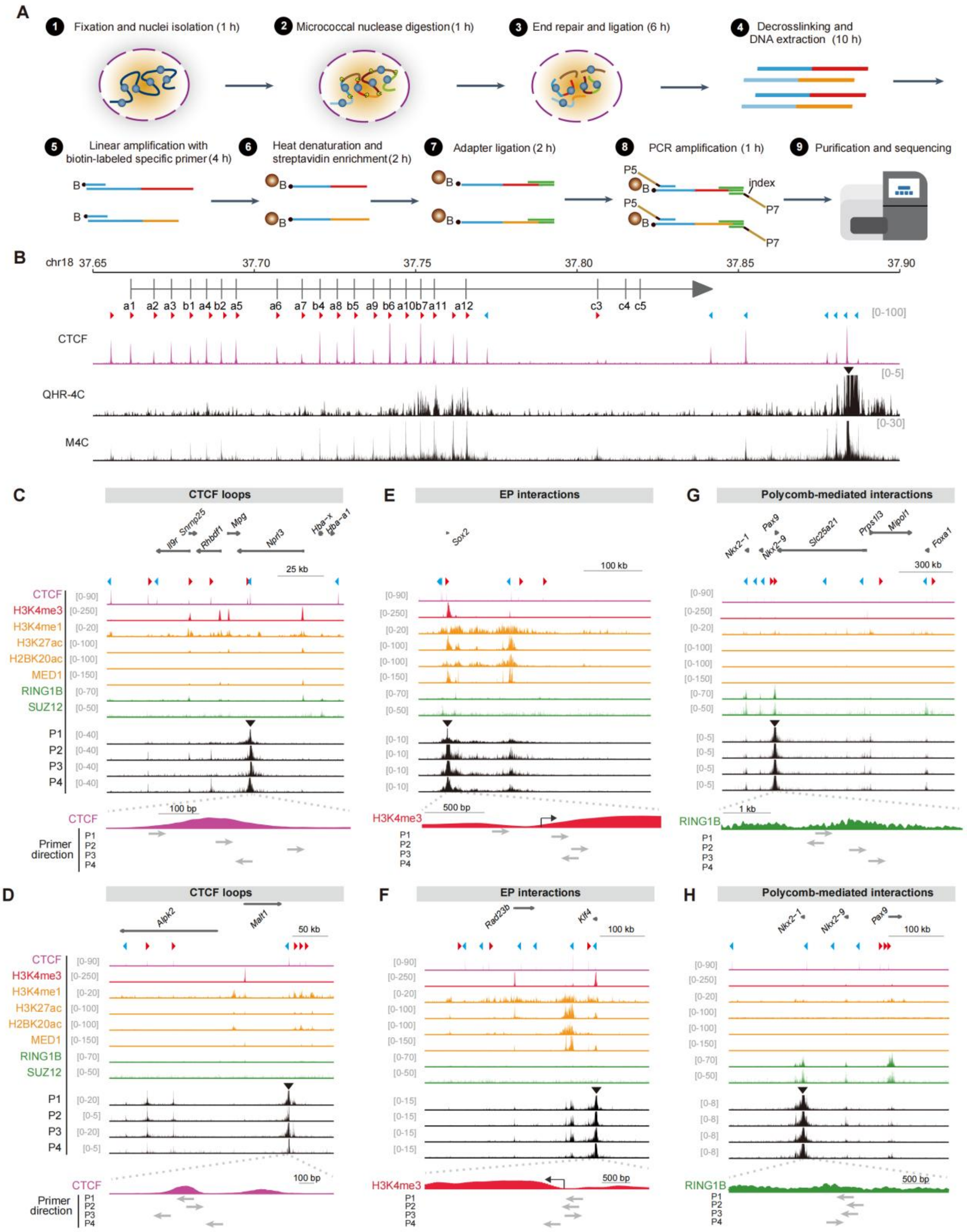
A
Fixation and nuclei isolation (1 h)
Micrococcal nuclease digestion (1 h)
End repair and ligation (6 h)
Decrosslinking and DNA extraction (10 h)
Linear amplification with biotin-labeled specific primer (4 h)
Heat denaturation and streptavidin enrichment (2 h)
Adapter ligation (2 h)
PCR amplification (1 h)
Purification and sequencing
B
chr18
CTCF
QHR-4C
M4C
C
CTCF loops
D
CTCF loops
E
EP interactions
F
EP interactions
G
Polycomb-mediated interactions
H
Polycomb-mediated interactions
CTCF
H3K4me3
H3K4me1
H3K27ac
H2BK20ac
MED1
RING1B
SUZ12
Primer direction

Figure S3. Development of MNase based 4C-seq. (A) The workflow of MNase based 4C-seq. (B) Genomic tracks showing CTCF signal, QHR-4C data, and M4C data at the *Pcdh* locus (chr18:37.65-37.90 Mb). The black triangle points to the anchor region. The red triangles represent CTCF motifs with forward direction, blue triangles represent CTCF motifs with reverse direction. (C-H) Genomic tracks showing CTCF, active chromatin markers (H3K4me3, H3K4me1, H3K27ac, H2BK20ac, MED1), repressive chromatin markers (RING1B, SUZ12), and M4C signal at a-*globin* (C chr11: 32.16-32.29 Mb), *Malt1* (D chr18: 65.25-65.55 Mb), *Sox2* (E chr3: 34.6-35.0 Mb), *Klf4* (F chr4: 55.15-55.65 Mb), *Pax9* (G chr12: 56.4-57.7 Mb) and *Nkx2-1* (H chr12: 56.399-56.800 Mb) loci. Black triangle represents the anchor regions that are enlarged at the bottom. Arrows represent the position and direction of specific primers. The red or blue triangles in B-H represent CTCF motifs with forward or reverse direction, respectively.

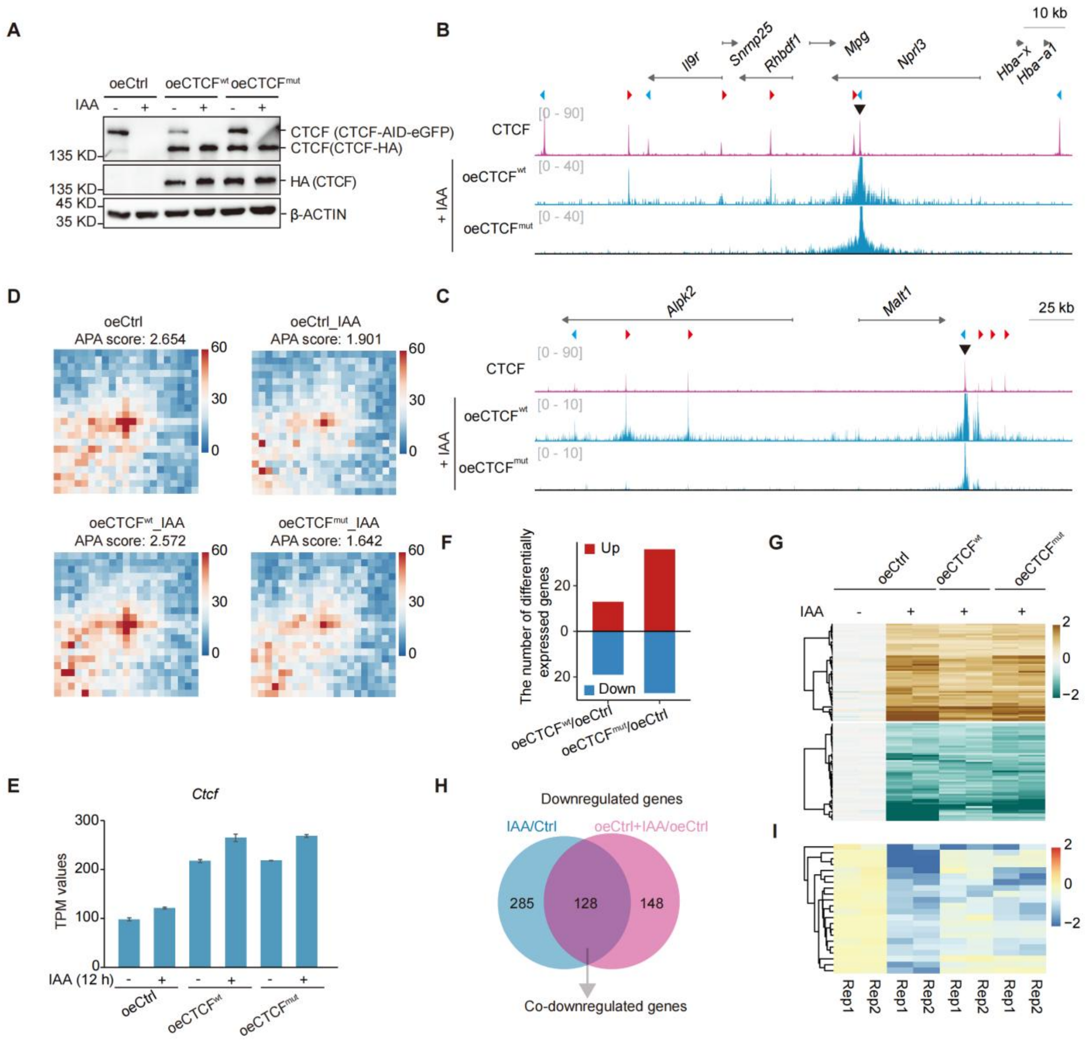
A
oeCtrl
oeCTCF^wt
oeCTCF^mut
IAA
CTCF (CTCF-AID-eGFP)
CTCF(CTCF-HA)
135 KD
HA (CTCF)
135 KD
45 KD
35 KD
β-ACTIN
B
10 kb
Il9r
Snrnp25
Rhbdf1
Mpg
Nprl3
Hba-x
Hba-a1
CTCF
[0 - 90]
+ IAA
oeCTCF^wt
[0 - 40]
oeCTCF^mut
[0 - 40]
C
Alpk2
Malt1
25 kb
CTCF
[0 - 90]
oeCTCF^wt
[0 - 10]
oeCTCF^mut
[0 - 10]
D
oeCtrl
APA score: 2.654
oeCtrl_IAA
APA score: 1.901
oeCTCF^wt_IAA
APA score: 2.572
oeCTCF^mut_IAA
APA score: 1.642
60
30
0
E
Ctcf
TPM values
300
200
100
0
IAA (12 h)
oeCtrl
oeCTCF^wt
oeCTCF^mut
F
The number of differentially expressed genes
Up
Down
20
0
20
oeCTCF^wt/oeCtrl
oeCTCF^mut/oeCtrl
G
oeCtrl
oeCTCF^wt
oeCTCF^mut
IAA
2
0
−2
H
Downregulated genes
IAA/Ctrl
oeCtrl+IAA/oeCtrl
285
128
148
Co-downregulated genes
I
2
0
−2
Rep1
Rep2
Rep1
Rep2
Rep1
Rep2
Rep1
Rep2

Figure S4. The effect of overexpression of CTCF^{wt} or CTCF^{mut} on gene expression in CTCF^{AID} mESCs. (A) Western blot detecting the exogenous expression level of CTCF^{wt} and CTCF^{mut} by CTCF and HA antibodies. (B and C) Genomic tracks of M4C data showing CTCF at a-*globin* and *Malt1* loci. The red or blue triangles represent CTCF motifs with forward or reverse direction, respectively. (D) Aggregate plots showing BL-Hi-C signal around CTCF loops in cells with different treatments. (E) Barplots showing the averaged transcripts per million (TPM) values of *Ctcf* genes from RNA-seq datasets in cells with different treatments. (F) Barplots showing the number of DEGs after exogenous expression of either CTCF^{wt} or CTCF^{mut}. (G) Heatmap showing expression pattern of genes that were consistently downregulated or upregulated following endogenous CTCF degradation in cells with different treatments. (H) Overlap analysis of DEGs following CTCF degradation in CTCF^{AID} mESCs (Fig. S1C) and oeCtrl CTCF^{AID} mESCs (Fig. 1G). (I) Heatmap showing expression pattern of genes, derived from Fig. 1H, that are downregulated upon CTCF degradation and have no CTCF binding at their promoters.

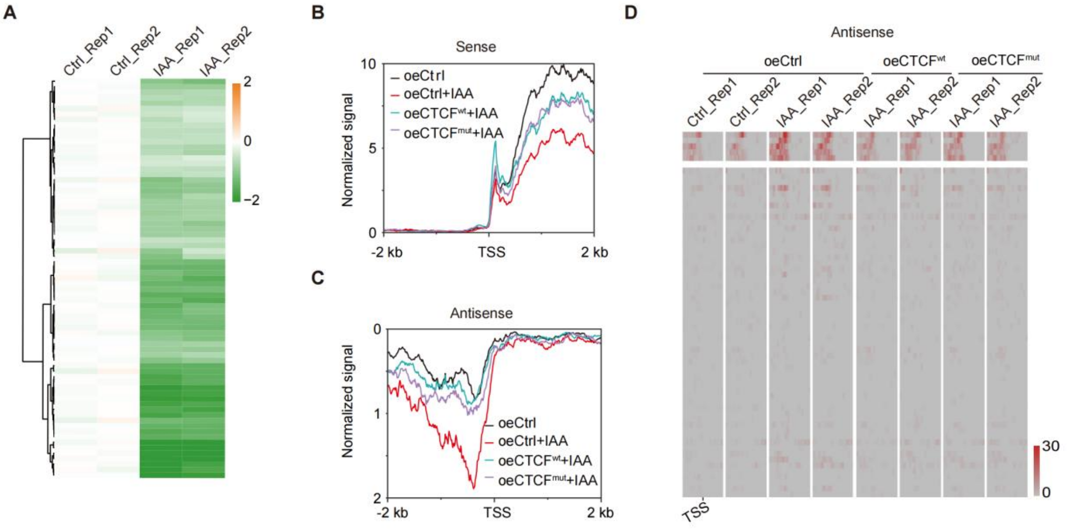

Figure S5. Transcriptional dynamics following CTCF depletion revealed by TT-seq. (A) Heatmap showing expression pattern of downregulated genes derived from Fig. 1I in TT-seq data. (B) Averaged expression level of downregulated genes in mESCs with different treatments. (C) Averaged antisense RNA expression level of downregulated genes in mESCs with different treatments. (D) Heatmap showing the detailed antisense RNA expression level of downregulated genes in mESCs with different treatments.

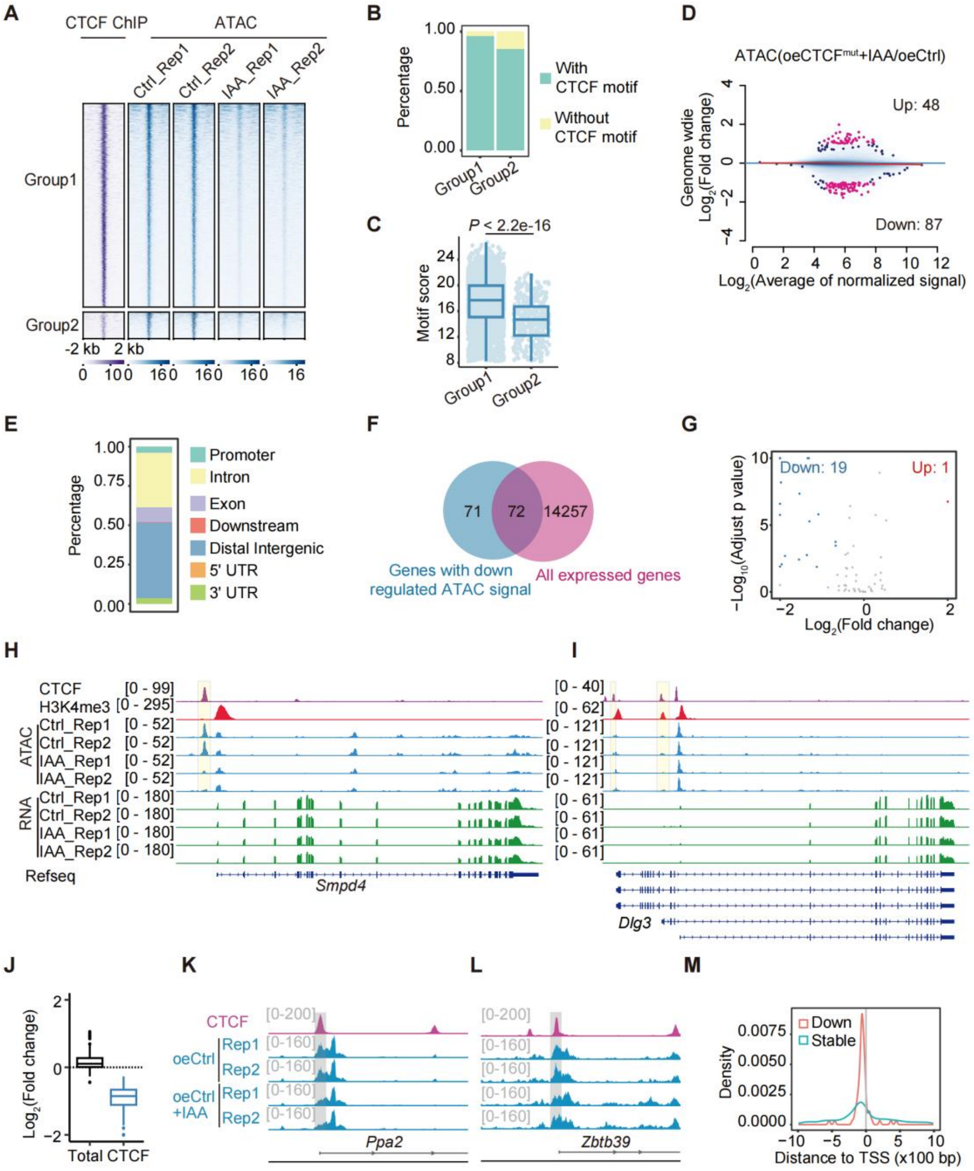

Figure S6. The relationship among CTCF binding, decreased chromatin accessibility and transcriptional changes upon CTCF degradation. (A) Heatmaps showing CTCF ChIP signals and ATAC signals in decreased chromatin accessible sites. (B) Barplots showing the percentage of ATAC sites containing CTCF motifs. (C) Boxplots showing the distribution of CTCF motif scores in two groups of ATAC sites. (D) Scatter plots showing the change of genome-wide ATAC signals between oeCtrl and oeCTCF^{mut} plus endogenous

CTCF depletion mESCs. (E) Bar plot showing gene annotation of decreased accessible sites. (F) Venn plot showing the overlap between genes with decreased ATAC signals at promoters and genes detected in the RNA-seq data. (G) Scatter plot showing gene expression changes following CTCF degradation. The genes used in this plot correspond to the overlapped genes in panel F. (H and I) Genomic tracks showing CTCF ChIP signals, ATAC signals and RNA-seq signals at *Smpd4* gene loci (H, chr16:17,616,136-17,645,078) and *Dlg3* gene loci (I, chrX:100,765,722-100,820,410). (J) Box plots representing the fold change of ATAC signals at promoters of downregulated genes upon CTCF degradation. “Total” means the overall ATAC signals, calculated using identified ATAC peaks, “CTCF” means the ATAC signals, calculated using 300 bp windows centered on CTCF binding sites. (K and L) Genomic tracks showing CTCF binding and ATAC signals at both the *Ppa2* promoter (panel K, chr3: 133,308,000-133,316,000) and *Zbtb39* promoter (panel L, chr10: 127,736,000-127,745,000), respectively. (M) For stable or downregulated genes with CTCF binding at their promoters, the distance between the center of CTCF motif and the transcription start site (TSS) is calculated, and the distribution of these distances is represented as line plots.

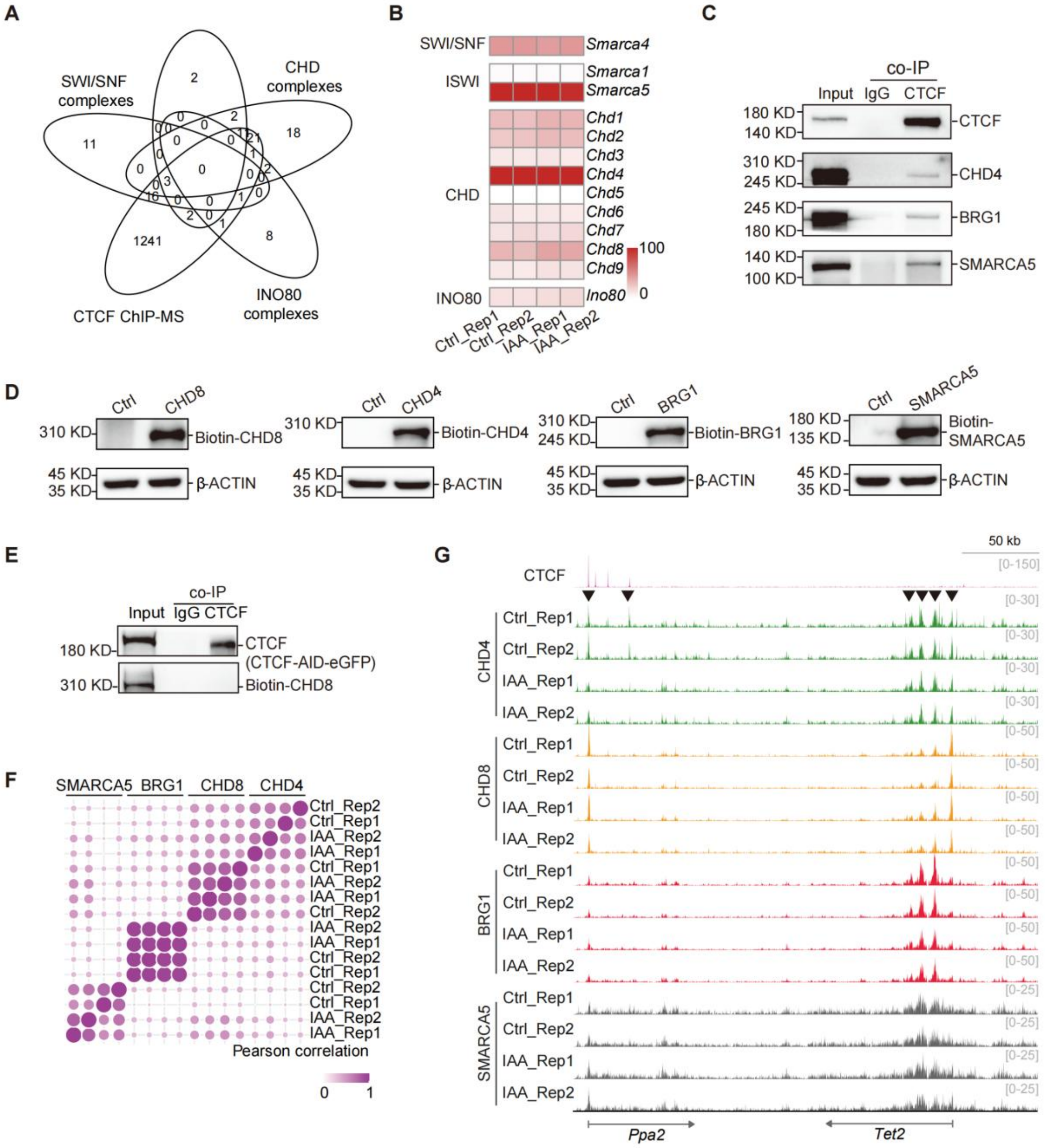

A
SWI/SNF complexes
CHD complexes
CTCF ChIP-MS
INO80 complexes
B
SWI/SNF
ISWI
CHD
INO80
Smarca4
Smarca1
Smarca5
Chd1
Chd2
Chd3
Chd4
Chd5
Chd6
Chd7
Chd8
Chd9
Ino80
Ctrl_Rep1
Ctrl_Rep2
IAA_Rep1
IAA_Rep2
C
co-IP
Input
IgG
CTCF
CHD4
BRG1
SMARCA5
D
Biotin-CHD8
Biotin-CHD4
Biotin-BRG1
Biotin-SMARCA5
β-ACTIN
E
CTCF (CTCF-AID-eGFP)
Biotin-CHD8
F
Pearson correlation
G
50 kb
Ppa2
Tet2

Figure S7. The relationship between CTCF and chromatin remodeling complexes. (A) Overlap analysis among proteins captured by CTCF ChIP-MS and the subunits of different chromatin remodeling complexes. (B) Heatmap showing the expression level of selected core subunits of chromatin remodeling complexes in mESCs. (C) Co-IP showing the interactions of CTCF with CHD4, BRG1, and SMARCA5, respectively. (D) Western blot analysis of the protein level of endogenous BIOTIN-tagged CHD4, CHD8, BRG1, and SMARCA5, respectively. (E) Co-IP showing protein interactions between CTCF and CHD8. (F) Correlation analysis among biotin-tagged CHD4, CHD8, SMARCA4, and SMARCA5 ChIP-seq datasets. (G) Genomic tracks showing the binding patterns of CTCF, CHD4, CHD8, BRG1 and SMARCA5 at *Ppa2* locus (chr3: 133,300,000-133,600,000). The Black arrows point to the binding sites of CHD4, CHD8, BRG1 or SMARCA5.

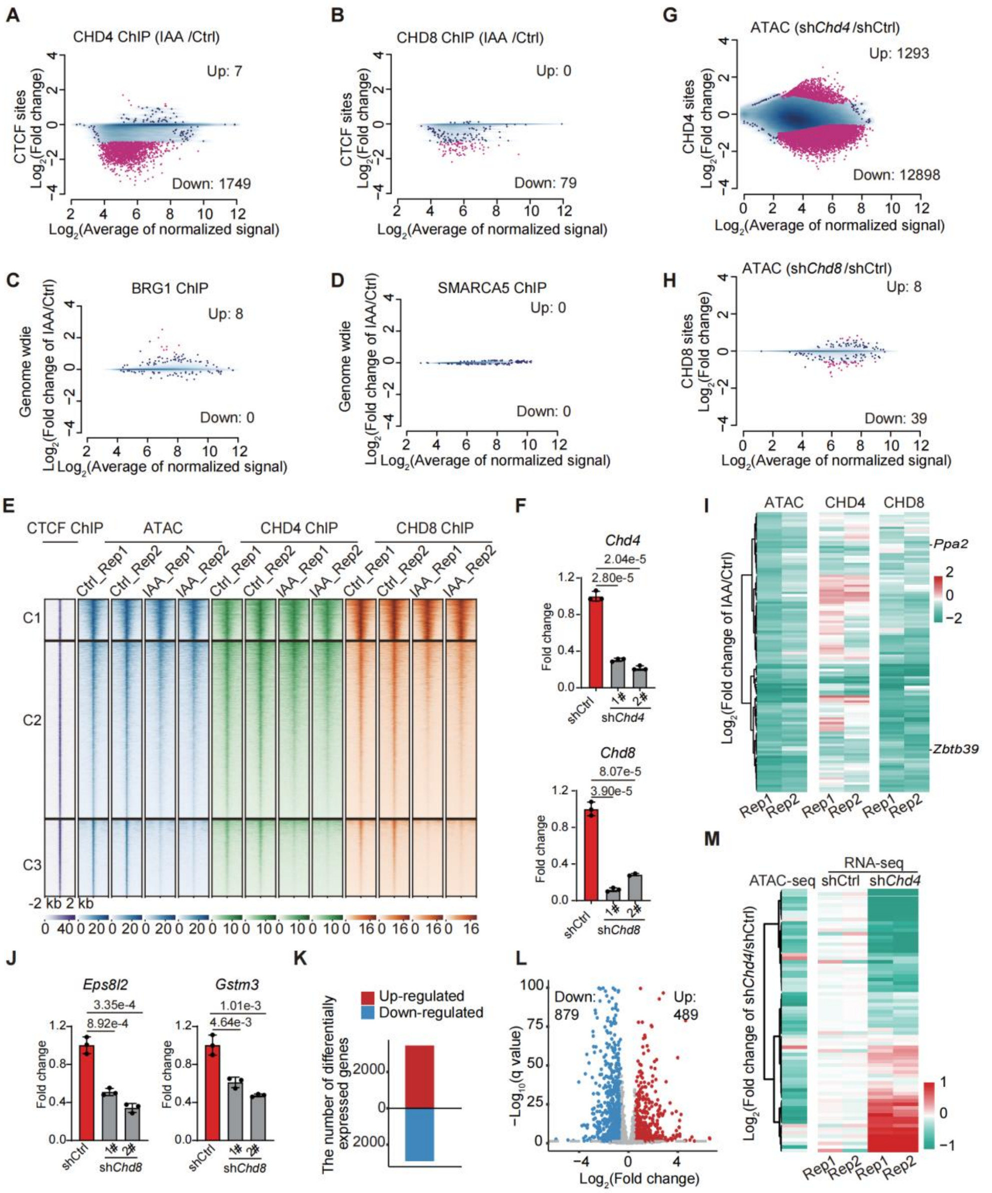

Figure S8. CTCF enhances chromatin accessibility through the promotion of CHD4 and CHD8 binding. (A and B) Scatter plots showing the enrichment of CTCF-overlapped CHD4 (panel A) or CHD8 (panel B) in untreated and IAA-treated CTCF^{AID} mESCs. (C and D) Scatter plots showing the enrichments of genome-wide BRG1 (SMARCA4) (panel C) and SMARCA5 (panel D) following CTCF degradation. (E) Heatmaps showing the changes in enrichment of CTCF, CHD4, CHD8, and ATAC signal at CTCF sites in

untreated and IAA-treated $CTCF^{AID}$ mESCs. (F) RT-qPCR analysis of the expression level of *Chd4* or *Chd8* after knockdown. Qualitative data are presented as mean ± SD and *P* values are calculated by two-sided unpaired *t*-test (n=3 independent biological replicates). (G) Scatter plots showing the changes of chromatin accessibility at ATAC sites overlapping with CHD4 binding sites after knockdown of *Chd4.* (H) Scatter plots showing the changes of chromatin accessibility at ATAC sites overlapping with CHD8 binding sites after knockdown of *Chd8*. (I) Heatmaps displaying the change of ATAC signals, CHD4 and CHD8 ChIP signals at the promoters of the overlapped genes from Fig.1H in untreated and IAA-treated $CTCF^{AID}$ mESCs. (J) RT-qPCR analysis of the expression level of *Eps8l2* and *Gstm3* after *Chd8* knockdown. Qualitative data are presented as mean ± SD and *P* values are calculated by two-sided unpaired *t*-test (n=3 independent biological replicates). (K) The number of differentially expressed genes after *Chd4* knockdown. (L) Scatter plot showing the expression changes of genes with reduced chromatin accessibility at their promoters following *Chd4* knockdown. (M) Heatmap showing changes in promoter ATAC signals and gene expression of CTCF target genes following *Chd4* knockdown.

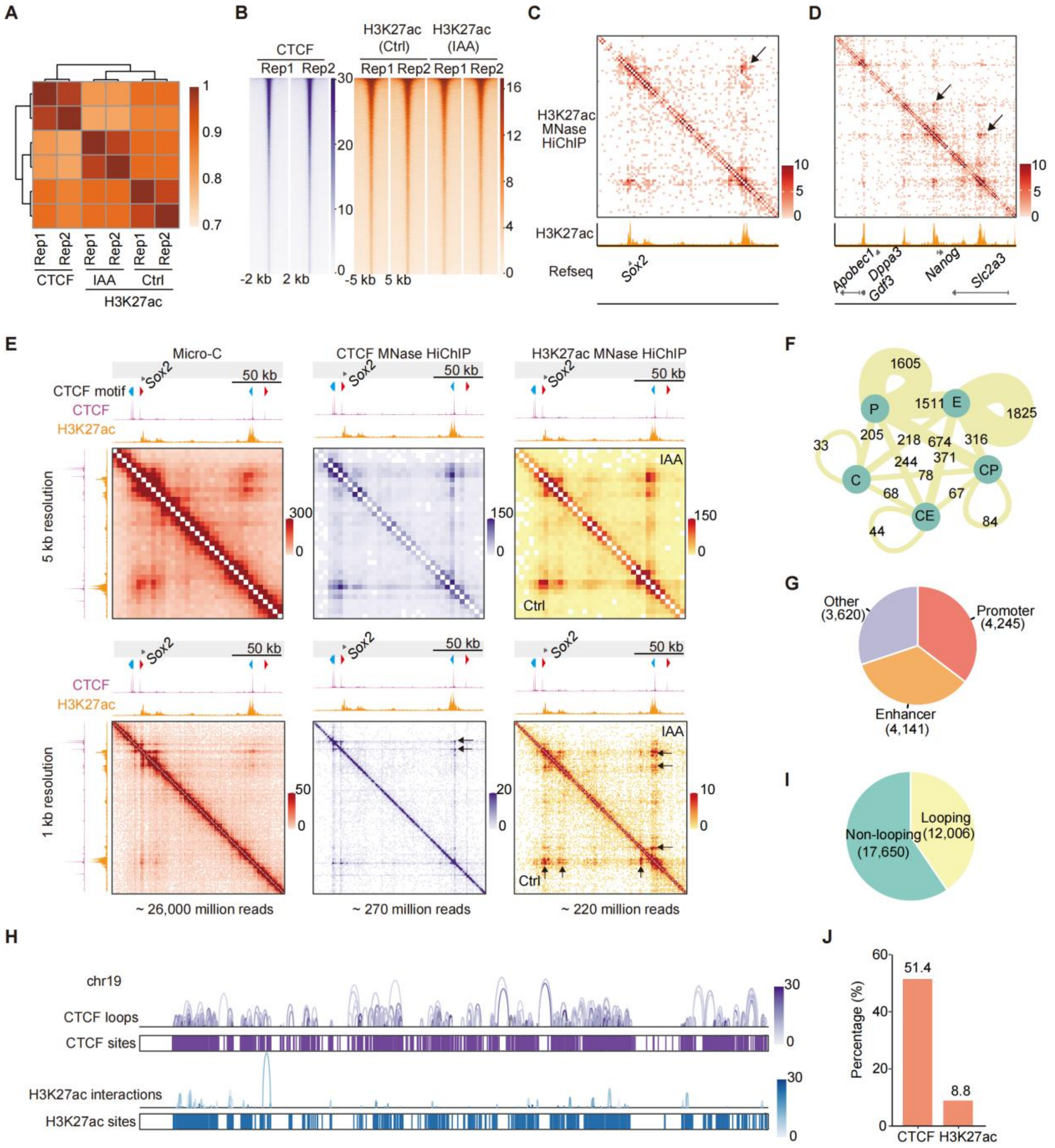
A
B
C
D
E
F
G
H
I
J
Rep1
Rep2
CTCF
IAA
Ctrl
H3K27ac
1
0.9
0.8
0.7
CTCF
Rep1 Rep2
H3K27ac (Ctrl)
H3K27ac (IAA)
Rep1 Rep2 Rep1 Rep2
30
20
10
0
16
12
8
4
0
-2 kb 2 kb
-5 kb 5 kb
H3K27ac MNase HiChIP
H3K27ac
Refseq
Sox2
10
5
0
Apobec1
Dppa3
Gdf3
Nanog
Slc2a3
Micro-C
CTCF MNase HiChIP
H3K27ac MNase HiChIP
CTCF motif
CTCF
H3K27ac
50 kb
5 kb resolution
1 kb resolution
300
150
50
20
IAA
Ctrl
~ 26,000 million reads
~ 270 million reads
~ 220 million reads
1605
1511
1825
P
E
C
CP
CE
33
205
218
674
316
244
371
78
68
67
44
84
Other (3,620)
Promoter (4,245)
Enhancer (4,141)
Looping (12,006)
Non-looping (17,650)
chr19
CTCF loops
CTCF sites
H3K27ac interactions
H3K27ac sites
Percentage (%)
60
40
20
51.4
8.8
CTCF
H3K27ac

Figure S9. Features of chromatin loops revealed by CTCF and H3K27ac MNase HiChIP. (A) Correlation analysis between CTCF and H3K27ac MNase HiChIP datasets. Correlation is calculated with HiCRep. (B) Heatmaps showing the enrichments of CTCF and H3K27ac derived from MNase HiChIP datasets. (C and D) H3K27ac-mediated chromatin interactions at *Sox2* (C) and *Nanog* (D) loci with 10% of final sequencing depth (approximate 20 million reads). Black arrows point to H3K27ac-mediated chromatin interactions. (E) Genomic tracks showing ChIP signals captured by CTCF and H3K27ac MNase HiChIP, and heatmaps showing chromatin interactions captured by Micro-C and MNase HiChIP with 5 kb or 1 kb resolutions at the *Sox2* locus (chr3: 34,620,000-34,790,000). Black arrows point to CTCF loops or H3K27ac interactions. (F) Networks displaying the types and numbers of H3K27ac interactions. Interactions mediated by the anchor types other than CTCF, promoter, enhancer were not shown. (G) Pie chart showing the percentage of promoters and enhancers at the anchor of H3K27ac interactions. (H) Schematic diagrams of CTCF sites, H3K27ac sites, and their mediated chromatin interactions across chromosome 19. (I) Pie chart showing the percentage of H3K27ac sites mediating chromatin interactions (looping) or not mediating chromatin interactions (non-looping). (J) Percentage of genome covered by CTCF loops or H3K27ac interactions.

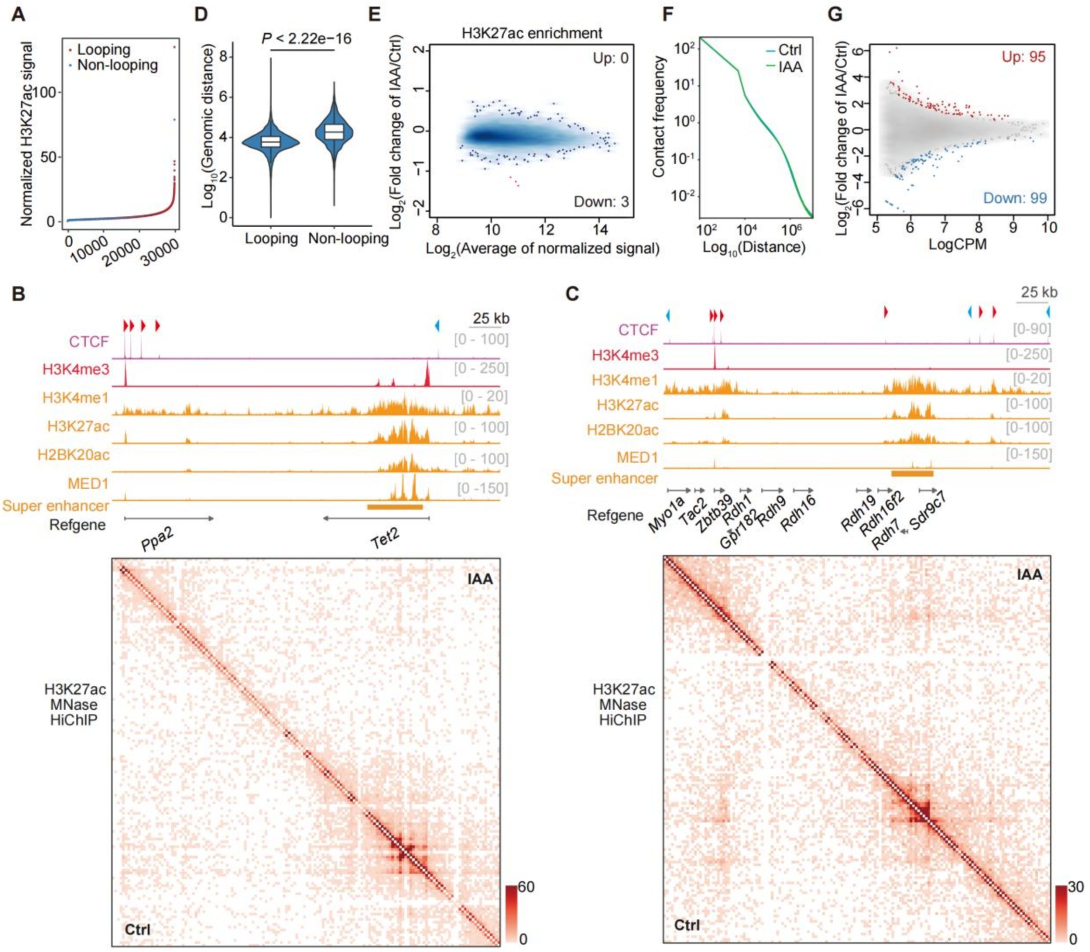

A
Normalized H3K27ac signal
Looping
Non-looping
D
P < 2.22e−16
Log10(Genomic distance)
Looping
Non-looping
E
H3K27ac enrichment
Up: 0
Down: 3
Log2(Fold change of IAA/Ctrl)
Log2(Average of normalized signal)
F
Contact frequency
Ctrl
IAA
Log10(Distance)
G
Up: 95
Down: 99
Log2(Fold change of IAA/Ctrl)
LogCPM
B
25 kb
CTCF
H3K4me3
H3K4me1
H3K27ac
H2BK20ac
MED1
Super enhancer
Refgene
Ppa2
Tet2
H3K27ac MNase HiChIP
IAA
Ctrl
C
25 kb
CTCF
H3K4me3
H3K4me1
H3K27ac
H2BK20ac
MED1
Super enhancer
Refgene
Myo1a
Tac2
Zbtb39
Rdh1
Gpr182
Rdh9
Rdh16
Rdh19
Rdh16f2
Rdh7
Sdr9c7
H3K27ac MNase HiChIP
IAA
Ctrl

Figure S10. The effect of CTCF degradation on H3K27ac-mediated chromatin interactions. (A) Scatter plot of normalized H3K27ac signals for looping or non-looping H3K27ac sites. H3K27ac signals were ranked by their strength. (B and C) Genomic tracks and heatmap showing the enrichments of CTCF, histone markers, and H3K27ac interactions in untreated and IAA-treated $CTCF^{AID}$ mESCs at both the *Ppa2* (panel B) and *Zbtb39* (panel C) loci. The red or blue triangles represent CTCF motifs with forward or reverse direction, respectively. (D) Violin plots overlaid with boxplots showing the genomic distance distribution of H3K27ac interactions and the genomic distance distribution between non-looping H3K27ac sites and their closest H3K27ac sites. The centre line indicates the median, the box represents the data between the first and third quantiles, the whiskers indicate the 1.5 interquartile range, and outliers are omitted. *P* values were calculated by a two-sided Mann-Whitney *U*-test. (E) Scatter plot showing the change of H3K27ac signal in untreated and IAA-treated $CTCF^{AID}$ mESCs. (F) The distance decay curves of H3K27ac MNase HiChIP data in untreated and IAA-treated $CTCF^{AID}$ mESCs. (G) Scatter plot showing the changes of H3K27ac interactions in untreated and IAA-treated $CTCF^{AID}$ mESCs.

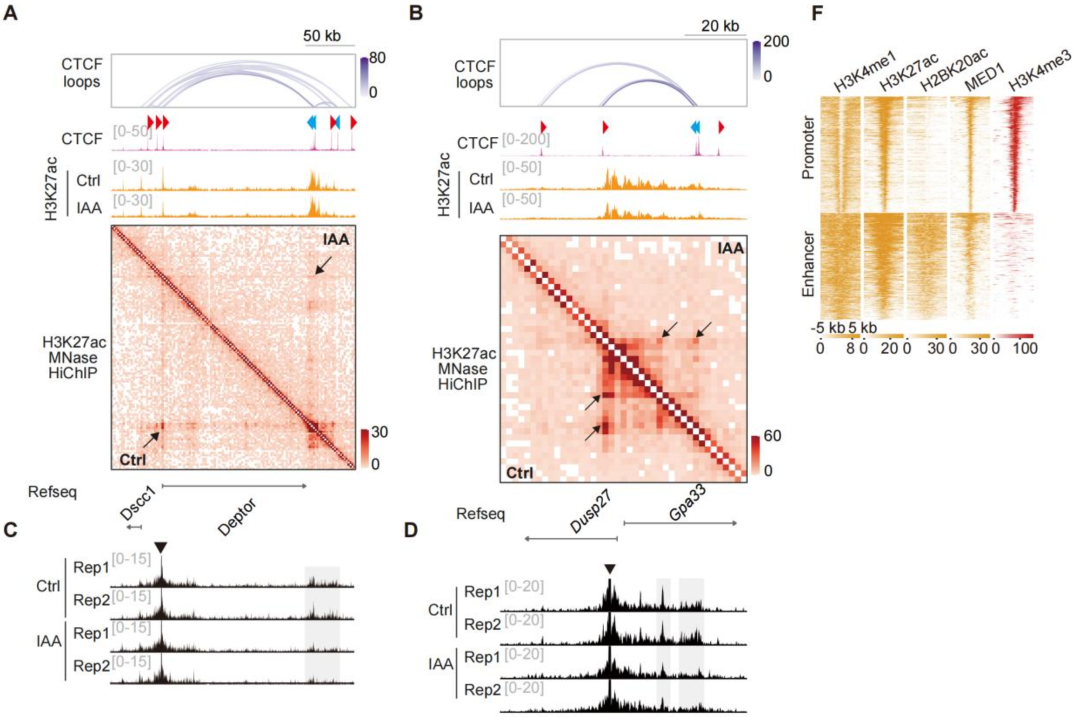

E

State (Emission)	H3K4me1	H2BK20ac	H3K27ac	MED1	H3K4me3	H3K27me3	H3K9me3	CTCF
Enhancer_nearby	0.05	0.38	0.01	0.00	0.00	0.00	0.01	0.00
Weak_enhancer1	0.84	0.77	0.05	0.01	0.00	0.00	0.00	0.01
Enhancer	0.81	0.98	0.93	0.14	0.11	0.00	0.02	0.01
Weak_enhancer2	0.55	0.02	0.44	0.22	0.02	0.00	0.01	0.03
H3K4me1	0.53	0.02	0.01	0.00	0.00	0.00	0.00	0.00
None	0.00	0.00	0.00	0.00	0.00	0.00	0.00	0.00
Bivalent_promoter	0.52	0.07	0.01	0.06	0.42	0.78	0.02	0.05
H3K27me3	0.01	0.00	0.00	0.00	0.00	0.48	0.00	0.00
H3K9me3	0.00	0.00	0.00	0.00	0.00	0.00	0.53	0.00
CTCF	0.14	0.01	0.00	0.01	0.02	0.00	0.00	0.98
Weak_promoter	0.58	0.03	0.15	0.02	0.95	0.00	0.00	0.02
Promoter	0.13	0.08	0.72	0.66	0.98	0.00	0.00	0.10

Figure S11. Characterization of the influence of CTCF loops on H3K27ac-mediated interactions. (A) Genomic tracks and heatmap of CTCF loops, CTCF ChIP signal, H3K27ac ChIP signal, and H3K27ac interactions at *Deptor* locus (chr15: 55,060,000-55,310,000). (B) Genomic tracks showing CTCF loops and enrichments of CTCF and H3K27ac, alongside a heatmap displaying H3K27ac interactions from H3K27ac MNase HiChIP data in untreated and IAA-treated $CTCF^{AID}$ mESCs at the *Dusp27* locus (chr1: 166,090,000-166,170,000). (C) Genomic tracks of M4C tracks showing chromatin interactions mediated by *Deptor* promoter in untreated and IAA-treated $CTCF^{AID}$ mESCs. The black triangle represents the anchor region. (D) Genomic tracks of M4C data showing the chromatin interactions mediated by the *Dusp27* promoter in untreated and IAA-treated $CTCF^{AID}$ mESCs. The black triangle represents the anchor region. (E) Genome segmentation by using different histone markers, MED1, and CTCF. (F) Heatmaps showing the enrichments of different histone markers at the H3K27ac-solo anchor of contain-type II H3K27ac interactions.

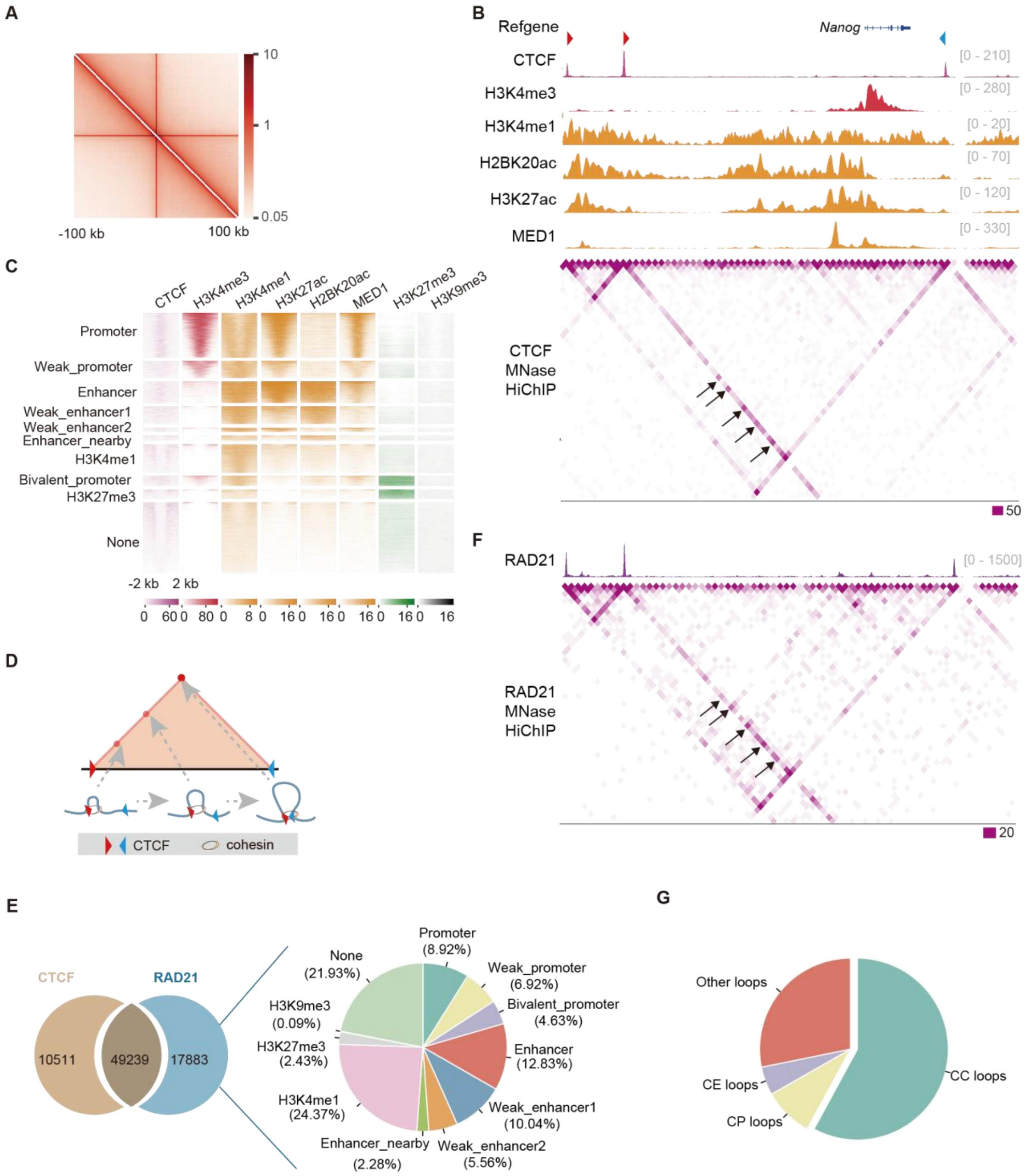
A
10
1
0.05
-100 kb
100 kb
B
Refgene
Nanog
CTCF
[0 - 210]
H3K4me3
[0 - 280]
H3K4me1
[0 - 20]
H2BK20ac
[0 - 70]
H3K27ac
[0 - 120]
MED1
[0 - 330]
CTCF
MNase
HiChIP
50
C
CTCF
H3K4me3
H3K4me1
H3K27ac
H2BK20ac
MED1
H3K27me3
H3K9me3
Promoter
Weak_promoter
Enhancer
Weak_enhancer1
Weak_enhancer2
Enhancer_nearby
H3K4me1
Bivalent_promoter
H3K27me3
None
-2 kb
2 kb
0 60 0 80 0 8 0 16 0 16 0 16 0 16 0 16
F
RAD21
[0 - 1500]
RAD21
MNase
HiChIP
20
D
CTCF
cohesin
E
CTCF
RAD21
10511
49239
17883
Promoter
(8.92%)
Weak_promoter
(6.92%)
Bivalent_promoter
(4.63%)
Enhancer
(12.83%)
Weak_enhancer1
(10.04%)
Weak_enhancer2
(5.56%)
Enhancer_nearby
(2.28%)
H3K4me1
(24.37%)
H3K27me3
(2.43%)
H3K9me3
(0.09%)
None
(21.93%)
G
Other loops
CE loops
CP loops
CC loops

Figure S12. CTCF enhances H3K27ac interactions when bound at loop anchors and forming same-directional loops. (A) Aggregate CTCF MNase HiChIP stripe signals mediated by CTCF sites. (B) Genomic tracks for the enrichments of CTCF, histone markers and heatmap for CTCF MNase HiChIP data at *Nanog* locus (chr6:122,660,100-122,730,100). Black arrows point to significant chromatin interactions in the stripes. (C) The enrichments of CTCF, MED1, and different histone markers in different types of chromatin regions. (D) Model depicting the formation of CTCF stripe-shaped contacts. (E) Venn plot (left panel) showing the overlap between CTCF sites and RAD21 sites derived from MNase HiChIP data. Pie plot (right panel) showing the genomic types of the RAD21 sites without CTCF binding. (F) Genomic track and heatmap of RAD21 MNase HiChIP data at *Nanog* locus. (G) Pie plot showing the types of RAD21-mediated chromatin interactions. CC, CP or CE loops represent CTCF-CTCF loops, CTCF-promoter loops or CTCF-enhancer loops, respectively.

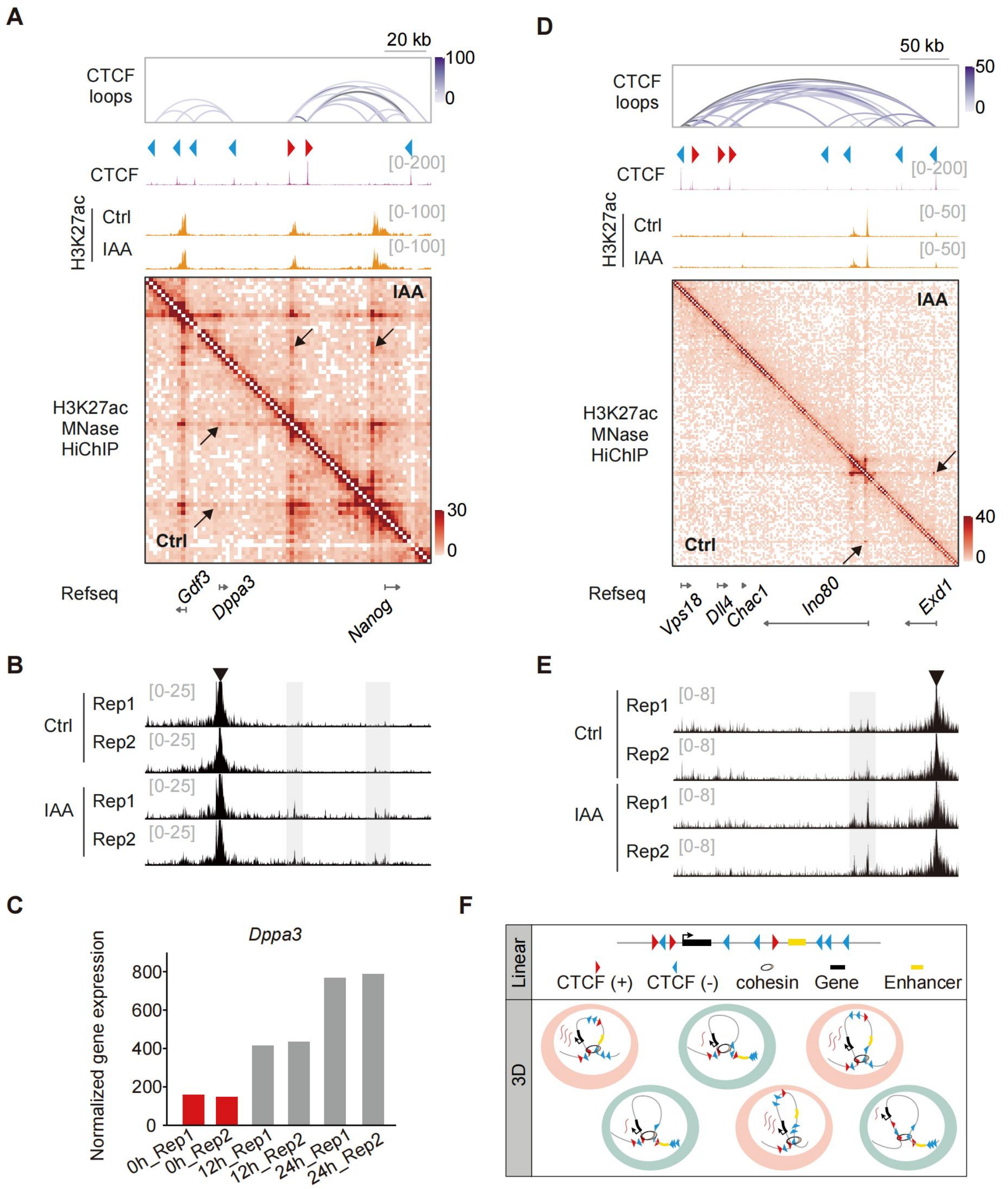

A
20 kb
CTCF loops
100
0
CTCF
[0-200]
H3K27ac
Ctrl
IAA
[0-100]
[0-100]
IAA
H3K27ac MNase HiChIP
30
0
Ctrl
Refseq
Gdf3
Dppa3
Nanog
D
50 kb
CTCF loops
50
0
CTCF
[0-200]
H3K27ac
Ctrl
IAA
[0-50]
[0-50]
IAA
H3K27ac MNase HiChIP
40
0
Ctrl
Refseq
Vps18
Dll4
Chac1
Ino80
Exd1
B
Ctrl
Rep1
Rep2
IAA
Rep1
Rep2
[0-25]
E
Ctrl
Rep1
Rep2
IAA
Rep1
Rep2
[0-8]
C
Dppa3
Normalized gene expression
800
600
400
200
0
0h_Rep1
0h_Rep2
12h_Rep1
12h_Rep2
24h_Rep1
24h_Rep2
F
Linear
CTCF (+)
CTCF (-)
cohesin
Gene
Enhancer
3D

Figure S13. Illustrations of EP interactions insulated by CTCF loops. (A and D) Genomic tracks displaying CTCF loops, enrichments of CTCF and H3K27ac, and heatmap showing H3K27ac interactions from MNase HiChIP data in untreated and IAA-treated $CTCF^{AID}$ mESCs at the *Dppa3* (A chr6: 122,590,000-122,730,000) and *Exd1* (D chr2: 119,280,000-119,570,000) loci, respectively. Black arrows point to the changed chromatin interactions. (B and E) Genomic tracks of M4C data showing the chromatin interactions mediated by either the *Dppa3* promoter (B) or the *Exd1* promoter (E) in untreated and IAA-treated $CTCF^{AID}$ mESCs. (C) Normalized gene expression level of *Dppa3* from RNA-seq data in untreated and IAA-treated $CTCF^{AID}$ mESCs. The red or blue triangles in panels A and D represent CTCF motifs with forward or reverse direction, respectively. (F) Model depicting a scenario of promotional and insulating effects of CTCF loops on gene expression.

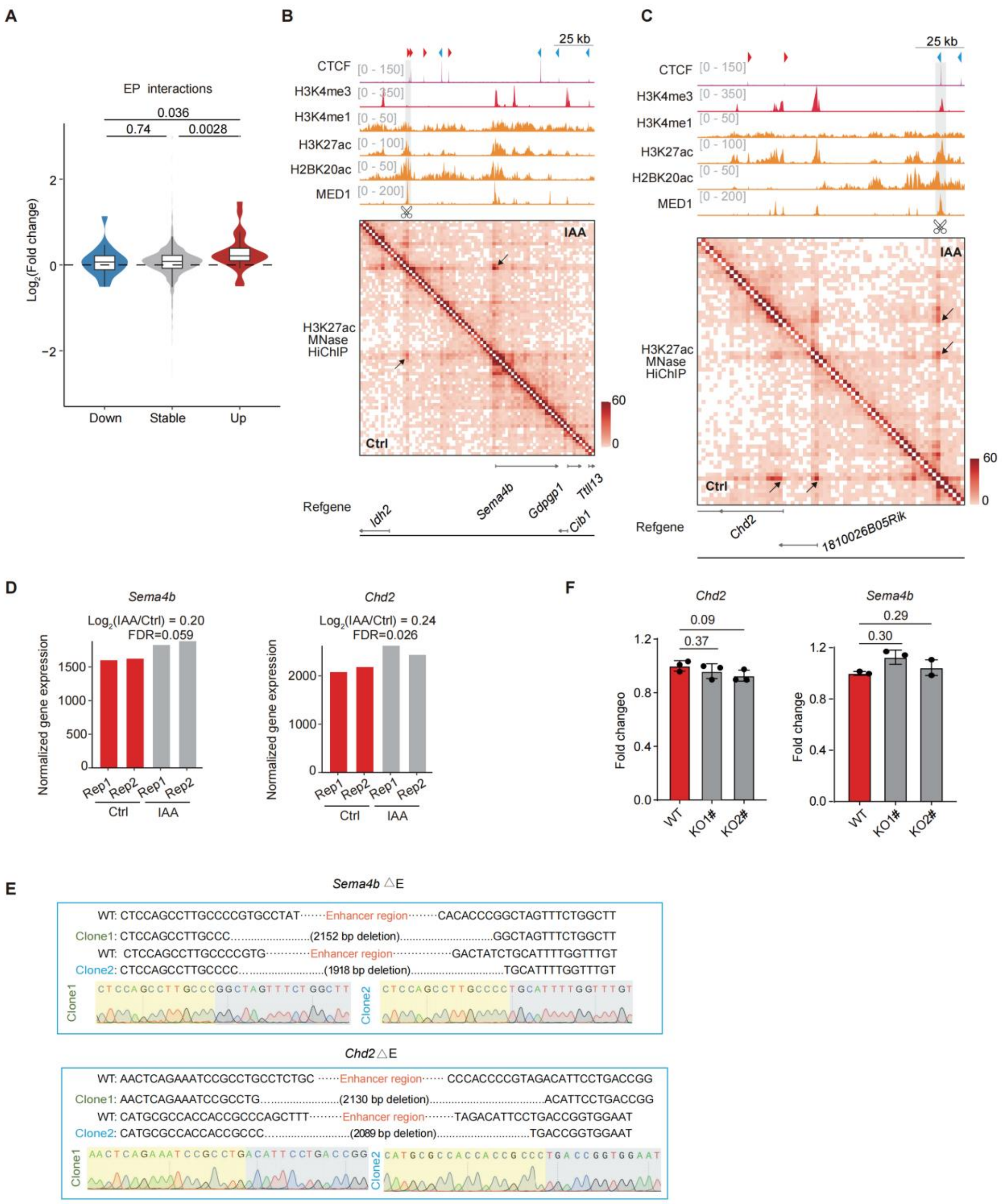
A
EP interactions
0.036
0.74
0.0028
Log2(Fold change)
Down
Stable
Up
B
25 kb
CTCF
H3K4me3
H3K4me1
H3K27ac
H2BK20ac
MED1
IAA
H3K27ac MNase HiChIP
Ctrl
Refgene
Idh2
Sema4b
Gdpgp1
Ttll13
Cib1
C
H3K27ac MNase HiChIP
Chd2
1810026B05Rik
D
Sema4b
Log2(IAA/Ctrl) = 0.20
FDR=0.059
Chd2
Log2(IAA/Ctrl) = 0.24
FDR=0.026
Normalized gene expression
Rep1
Rep2
Ctrl
IAA
F
Chd2
Sema4b
Fold changeo
Fold change
WT
KO1#
KO2#
0.09
0.37
0.29
0.30
E
Sema4b △E
WT: CTCCAGCCTTGCCCCGTGCCTAT·······Enhancer region·······CACACCCGGCTAGTTTCTGGCTT
Clone1: CTCCAGCCTTGCCC..........(2152 bp deletion)..........GGCTAGTTTCTGGCTT
WT: CTCCAGCCTTGCCCCGTG···········Enhancer region················GACTATCTGCATTTTGGTTTGT
Clone2: CTCCAGCCTTGCCCC..........(1918 bp deletion)..........TGCATTTTGGTTTGT
Chd2 △E
WT: AACTCAGAAATCCGCCTGCCTCTGC ······Enhancer region······ CCCACCCCGTAGACATTCCTGACCGG
Clone1: AACTCAGAAATCCGCCTG..........(2130 bp deletion)..........ACATTCCTGACCGG
WT: CATGCGCCACCACCGCCCAGCTTT·········Enhancer region········TAGACATTCCTGACCGGTGGAAT
Clone2: CATGCGCCACCACCGCCC..........(2089 bp deletion)..........TGACCGGTGGAAT

Figure S14. The impact of enhancer knockout on gene expression. (A) Violin plots overlaid with boxplots showing log2 fold change in expression of genes associated with different types of EP interactions upon CTCF degradation. The centre line indicates the median, the box represents the data between the first and third quantiles, the whiskers indicate the 1.5 interquartile range, and outliers are omitted. *P* values are calculated by a two-sided Mann-Whitney *U*-test. (B and C) Genomic tracks showing the enrichments of CTCF, histone markers and heatmap showing H3K27ac interactions at the *Sema4b* locus (panel B, chr7: 80,100,000-80,250,000) and *Chd2* locus (panel C, chr7: 73,500,000-73,630,000) in untreated and IAA-treated $CTCF^{AID}$ mESCs. Black arrows point to the changed chromatin interactions. The red or blue triangles represent CTCF motifs with forward or reverse direction, respectively. (D) Normalized gene expression level of *Sema4b* and *Chd2* from RNA-seq datasets in untreated and IAA-treated $CTCF^{AID}$ mESCs. Fold change and FDR values are obtained from differentially expression analysis of RNA-seq datasets. (E) Schematic and Sanger sequencing of genomic sequences for enhancer deletion at the *Sema4b* or *Chd2* gene loci, respectively. (F) RT-qPCR analysis showing gene expression of *Chd2* or *Sema4b* in untreated and IAA-treated $CTCF^{AID}$ mESCs. Quantitative data are presented as mean ± SD, *P* values are calculated by two-sided unpaired *t*-test (n=3 independent biological replicates).

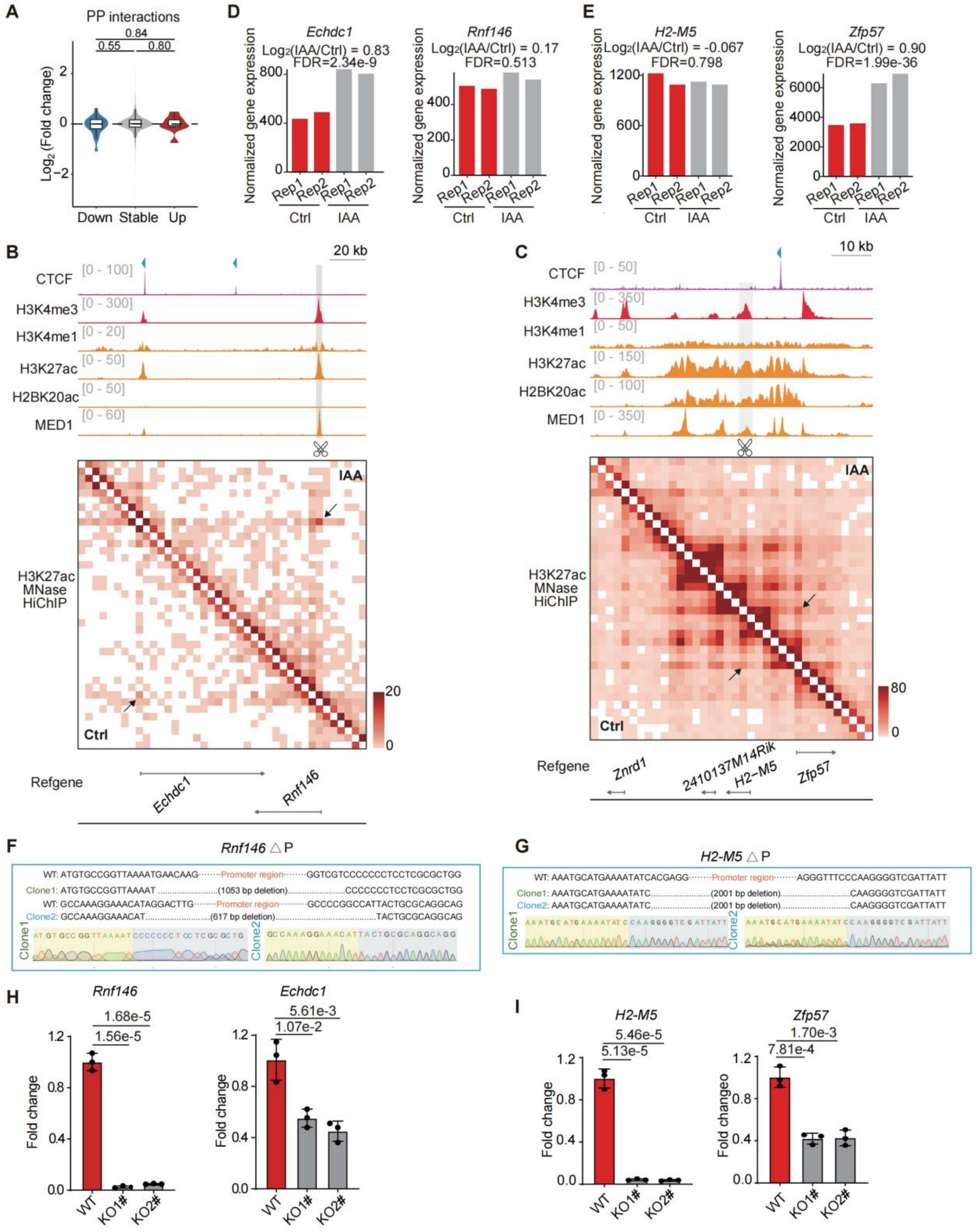

Figure S15. The impact of promoter knockout-mediated disruption of PP interactions on gene expression. (A) Violin plots overlaid with boxplots showing log2 fold change in expression of genes associated with different types of PP interactions upon CTCF degradation. The centre line indicates the median, the box represents the data between the first and third quantiles, the

whiskers indicate the 1.5 interquartile range, and outliers are omitted. *P* values are calculated by a two-sided Mann-Whitney *U*-test. (B) Genomic tracks showing the enrichments of CTCF, histone markers and heatmap showing H3K27ac interactions at the *Rnf146* locus (chr10: 29,295,000-29,375,000). (C) Genomic tracks showing the enrichments of CTCF and histone markers, and heatmap showing H3K27ac interactions in untreated and IAA-treated CTCF^{AID} mESCs at the *H2-M5* locus (chr17: 36,950,000-37,020,000). Black arrows point to the changed chromatin interactions. (D and E) Normalized expression level of *Echdc1*, *Rnf146* (panel D), *H2-M5* and *Zfp57* (panel E) from RNA-seq datasets in untreated and IAA-treated CTCF^{AID} mESCs. (F and G) Schematic and Sanger sequencing of genomic sequences in the *Rnf146* (panel F) or *H2-M5* (panel G) promoter deleted mESCs. (H) RT-qPCR analysis of the expression of *Rnf146* and *Echdc1* in control and *Rnf146* promoter knockout cells. (I) RT-qPCR analysis showing gene expression of *H2-M5* and *Zfp57* in control and *H2-M5* promoter deleted mESCs. In bar plots (D, E), the fold changes and FDR values are obtained from differential expression analysis of RNA-seq datasets. In bar plots (H, I), qualitative data are presented as mean ± SD and *P* values are calculated by two-sided unpaired *t*-test (n=3 independent biological replicates).

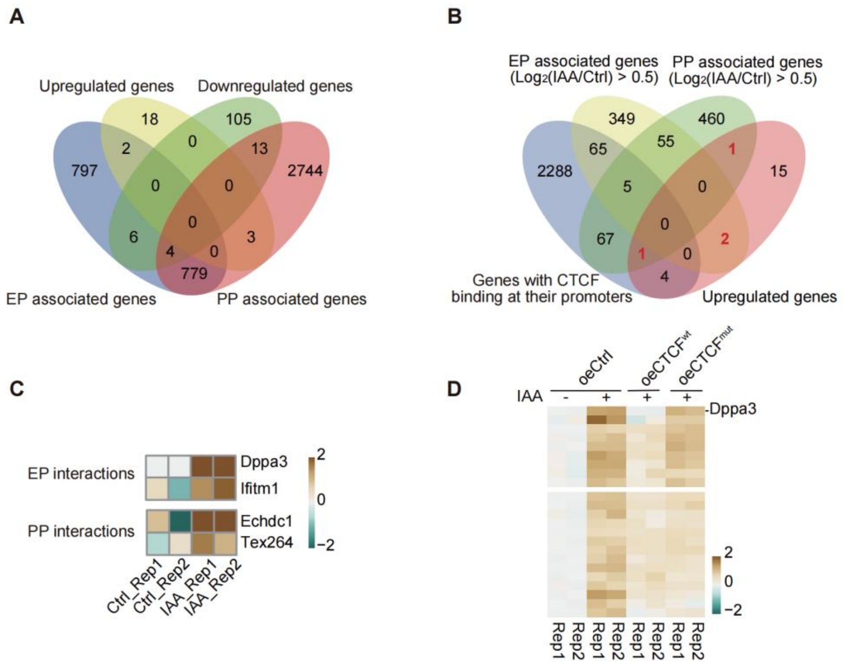

Figure S16. Functional comparison of CTCF loop-dependent and loop-independent roles in regulating gene expression. (A) Venn diagram illustrating the overlap among DEGs resulting from CTCF degradation, EP-associated genes, and PP-associated genes. (B) Venn diagram depicting the overlap among upregulated genes, genes with CTCF binding at their promoters and genes associated with upregulated EP or PP interactions. Upregulated genes that are linked to altered EP or PP interactions were highlighted in red. (C) Heatmaps displaying changes in EP or PP interactions and ATAC signal at promoters for overlapped upregulated EP- or PP-associated genes identified in panel B. (D) Heatmap showing expression changes of upregulated genes under different treatments.

III: Supplementary Methods

1. Detailed protocol for MNase-4C

Step1. Formaldehyde fixation and nuclei isolation

Cells were digested into single cell suspension by 0.25% Trypsin, counted using an automatic cell counter, and crosslinked with formaldehyde to a final concentration of 2% on a roller mixer at room temperature (RT) for 10 minutes (min). Glycine was added to a final concentration of 125 mM to quench the formaldehyde. The cells were then pelleted by centrifugation at 1000 x g at 4°C for 5 min, followed by washing with cold PBS. After washing, the cells were pelleted again by centrifugation and either stored at -80°C or processed for the following steps.

For nuclear preparation, the fixed cells were resuspended in 1 mL cold lysis buffer (10 mM Tris-HCl (pH 8.0), 10 mM NaCl, 0.2% IGEPAL CA-360, 1 × protease inhibitor cocktail) and incubated on ice for 20 min.

Step2. Micrococcal Nuclease (MNase) Digestion

Nuclei were pelleted by centrifugation at 2500 x g at 4°C for 5 min, then resuspended in 800 μL of MNase buffer with reduced-calcium-content (10 mM Tris-HCl (pH 7.5), 1 mM $CaCl_2$). A total of 50 Kunitz units of MNase (NEB#M0247S) were added to digest the chromatin. The reaction was incubated at 37°C for 30 min, then spined at 850 rpm on thermomixer. MNase digestion was stopped by adding EDTA to a final concentration of 5 mM,

followed by incubation at 65°C for 10 min to fully inactivate the enzyme.

Step3. End Repair of DNA Fragment

Briefly, 25 μL of ddH_2O, 5 μL of 10 × NEB buffer 2.1 (NEB#B7202S), 10 μL of 10 mM ATP (NEB#P0756L), 2.5 μL of T4 Polynucleotide Kinase (NEB#M0201L), 2.5 μL of 100 mM DTT were added to the pellet and incubated at 37°C for 15 min with intermittent mixing. Following this, 5 μL Klenow Fragment (NEB#M0210L) was added and incubated at 37°C for an additional 15 min with mixing at intervals. Next, 20.25 μL of ddH_2O, 2.5 μL of 10 × T4 DNA Ligase Buffer (NEB#B0202S), 0.25 μL of 20 mg/mL BSA (NEB#B9000), and 2 μL of dNTP Mix (NEB#N0447S) were added to the pellet and incubated at RT for 45 min with rotation. The reation was stopped by adding EDTA to a final concentration of 30 mM, and followed by inactivation at 65°C for 10 min. Finally, the samples were pelleted by centrifugation at 16000 x g at 4°C for 5 min and washed with cold buffer (50 mM Tris-HCl (pH 7.5), 10 mM $MgCl_2$).

Step4. Proximity Ligation

Proximity ligation was performed at 16°C with gentle rotation overnight in a total volume of 1 mL, containing 1 × T4 ligase buffer, 0.1 mg/mL BSA, and 4000 unit of T4 DNA ligase (NEB#M0202L).

Step5. Reverse cross-linking and DNA extraction

The chromatin was resuspended in 200 μL of DNA elution buffer (0.1 M $NaHCO_3$, 1% SDS, freshly prepared), followed by the addition of 4 μL 5 M NaCl and 2 μL RNase A. The mixture was incubated overnight at 65°C. The DNA was then purified using the MinElute PCR Purification Kit (Qiagen, Cat#28004), eluted in 100 μL of ddH_2O, and quantified using a Qubit fluorometer (Thermo Fisher Scientific).

Step6. Linear amplification reaction

A linear amplification step was applied to enrich for ligation events associated with a specific viewpoint using a 5'biotin-tagged primer. 500 ng of DNA was resuspended in 50 μL of 2 × Phanta buffer (Vazyme Biotech, Cat# P505), 1.6 μL of Phanta polymerase, 0.8 μL of 10 mM dNTPs, 1 μL Bio-primer (2 μM) and ddH_2O to a total volume of 100 μL. The following PCR program was performed: 95°C, 2 min; 95°C, 15 s; 58°C, 25 s; 72°C, 1 min for 110 cycles; and a final extension at 72°C for 5 min. The amplification products were denatured by heating at 95°C for 5 min and immediately cooled on ice to obtain single-stranded DNA (ssDNA).

Step7. Biotin capture

10 μL of M280 beads were washed twice with 200 μL of 1 × Binding and Washing (B&W) Buffer (5 mM Tris-HCl (pH 7.5), 0.5 mM EDTA, 1 M NaCl) and then incubated with 2% BSA for 30 min with rotation to reduce non-specific

binding. The beads were added to the samples and incubated at RT for 45 min with rotation. After capture, the beads were washed four times with 200 μL of 1 × B&W Buffer.

Step8. Adaptor ligation

18.5 μL of ddH_2O, 20 μL of 2 × T4 DNA ligase buffer, 1 μL of T4 DNA ligase (Rapid) (Vazyme Biotech, Cat# N103), 0.5 μL of 5 μM adaptor were added to the beads. The mixture was incubated at 30°C for 30 min with rotation.

Step9. Library construction

MNase-4C libraries were constructed using specific primer pairs. The forward primers contained Illumina P5 with an index and sequences near a specific viewpoint, while the reverse primers contained Illumina P7 with an index. After ligation, the beads were washed four times with 200 μL of 1 × B&W buffer and resuspended in 16 μL ddH_2O, 25 μL of 2 × Phanta buffer, 1 μL of Phanta polymerase, 1 μL of 10 mM dNTPs, 1 μL P5-primer, 1 μL P7-primer, 5 μL NTR short primers. The following PCR program was performed: 95°C, 2 min; 95°C, 15 s; 58°C, 25 s; 72°C, 1 min for 18-20 cycles; and a final extension at 72°C for 5 min. After PCR, libraries were placed on a magnet, eluted into new tubes, and then purified for sequencing. A 2% agarose gel was prepared, and the samples were separated by electrophoresis in TAE Buffer. DNA fragments ranging from 200–800 bp were excised, recovered using the NucleoSpin Gel

and PCR Clean-up kit (Macherey-nagel#740609.50), and prepared for high-throughput sequencing.

2. Detailed protocol for MNase HiChIP

Step1. Formaldehyde fixation

Cells were digested into single cell using 0.25% Trypsin, counted with an automatic cell counter, and crosslinked with formaldehyde to a final concentration of 1% on a roller mixer at room temperature (RT) for 10 minutes (min). Glycine was then added to a final concentration of 125 mM to quench the formaldehyde. The cells were pelleted by centrifugation at 1000 x g at 4°C for 5 min, washed with cold PBS, and pelleted again by centrifugation. The cell pellet was either stored at -80°C or used immediately for Step 2.

Step2. Lysis and MNase Digestion

Use 5 × 10^6 of cells in the following steps. The fixed cells were resuspended in 1 mL cold lysis buffer (10 mM Tris-HCl (pH 8.0), 10 mM NaCl, 0.2% IGEPAL CA-630, 1 × protease inhibitor cocktail) and incubated on ice for 20 min. Nuclei were by centrifugation at 2500 x g at 4°C for 5 min, then resuspended in 800 μL of reduced-calcium-content MNase buffer (10 mM Tris-HCl (pH 7.5), 1 mM $CaCl_2$). Next, 100 Kunitz U of MNase (NEB#M0247S) were added to digest the chromatin. The reaction was incubated at 37°C for 30 min at 850 rpm in a

thermomixer. MNase digestion was stopped by adding EDTA to a final concentration of 5 mM. The reaction was centrifuged at 2500 x g at 4°C for 5 min, and the digestion buffer was discarded.

Step3. End Repair of DNA Fragment

The following reagents were added to the pellet: 50 μL of ddH_2O, 10 μL of 10 × NEB buffer 2.1 (NEB#B7202S), 20 μL of 10 mM ATP(NEB#P0756L), 5 μL of T4 Polynucleotide Kinase (NEB#M0201L), 5 μL of 100 mM DTT. The mixture was incubated at 37°C for 15 min with intermittent mixing. Next, 10 μL Klenow Fragment (NEB#M0210L) was added and incubated at 37°C for an additional 15 min with intermittent mixing. Then, 33.75 μL of ddH_2O, 5 μL of 10 × T4 DNA Ligase Buffer (NEB#B0202S), 0.25 μL of 20 mg/mL BSA (NEB#B9000), 20 μL of 10 mM ATP, 0.5 μL of 10 mM dGTP (Invitrogen, 18254011), 0.5 μL of 10 mM dTTP (Invitrogen, 18255018), 5 μL of 1 mM Biotin-14-dATP (Jena Bioscience, NU-835-BIO14-L), 5 μL of 1 mM Biotin-11-dATP (Jena Bioscience, NU-1175-BIOX-L) were added to the pellet. The mixture was incubated at RT for 45 min with rotation. The reaction was stopped by adding EDTA to a final concentration of 30 mM and completely inactivated by incubating at 65°C for 10 min. Nuclei were pelleted by centrifugation at 2500 x g at 4°C for 5 min and washed with cold buffer (50mM Tris-HCl (pH 7.5), 10mM $MgCl_2$).

Step4. Proximity Ligation

Proximity ligation was performed at 16°C for 4 hours with slow rotation in a total volume of 1 mL containing 1 × T4 ligase buffer, 0.1 mg/mL BSA and 4000 unit of T4 DNA ligase (NEB#M0202L).

Step5. Sonication and chromatin immunoprecipitation

After proximity ligation, the nuclei were by centrifugation at 2500 x g for 5 min, and the supernatant was discarded. The nuclei were then resuspended in 100 µL nuclear Lysis Buffer (50 mM Tris-HCl (pH 7.5), 10 mM EDTA, 1% SDS, 1 × protease inhibitor cocktail) and sonicated using a Bioruptor Plus sonicator at 4°C under high-power for 6 cycles (30 s on/30 s off). After sonication, the samples were cleared by centrifugation at 16000 x g for 10 min, and the supernatant was collected. Clarified samples were transferred to Eppendorf tubes, and 900 µL of ChIP Dilution Buffer (0.01% SDS, 1.1% Triton X-100, 1.2 mM EDTA, 16.7 mM Tris-HCl pH 7.5, 167 mM NaCl) were added. Samples were precleared by adding 5 µL of protein A and 5 µL of protein G Dynabeads to each sample, followed by incubation for 1 h at 4°C with rotation.The tubes were placed on a magnetic stand, and the cleared supernatant was transferred to new tube. 2 µg of H3K27ac antibody (Active Motif #39133) were added to each tube (for 5 million cells) and incubated overnight at 4°C with rotation.15 µL of protein A and 15 µL of protein G Dynabeads were precleared by adding 1 mL of 1 mg/mL BSA and 1 mg/mL yeast transfer ribonucleic acid in ChIP dilution buffer and incubating for 1 h at 4°C with rotation. The preblocked

beads were washed twice with ChIP dilution buffer, added to sample, and rotated at 4°C for 3 h. After bead capture, the beads were washed twice each of the following buffers: low-salt wash buffer (0.1% SDS, 1% Triton X-100, 2 mM EDTA, 20 mM Tris-HCl (pH 8.0), 150 mM NaCl), high-salt wash buffer (0.1% SDS, 1% Triton X-100, 2 mM EDTA, 20 mM Tris-HCl (pH 8.0), 500 mM NaCl), LiCl wash buffer (10 mM Tris-HCl (pH 8.0), 250 mM LiCl, 1% NP-40, 1% sodium deoxycholate, 1 mM EDTA) and TE buffer (10 mM TrisHCl (pH 8.0), 1 mM EDTA).

Step6. DNA elution and reverse crosslinking

Sample beads were resuspended in 200 μL of DNA elution buffer (0.1 M NaHCO3, 1% SDS, freshly prepared). Then, 4 μL 5M NaCl and 2 μL of RNase A were added to each sample, and the mixture was incubated overnight at 65°C. Beads were placed on a magnet, and supernatant was transferred to a fresh tube. Next, 4 μL 20 mg/mL proteinase K was added, and the samples were incubated at 55°C for 1.5 h. After incubation, the samples were purified using the MinElute PCR Purification Kit (Cat.28004) and eluted in 20 μL of water. The ChIPed DNA was quantified using the Qubit system (Thermo Fisher Scientific).

Step7. Biotin Capture

10 μL of M280 beads were washed twice with 200 μL of 1 × Binding and

Washing (B&W) Buffer (5 mM Tris-HCl (pH 7.5), 0.5 mM EDTA, 1 M NaCl). The beads were then incubated with 2% BSA for 30 min with rotation to reduce non-specific binding. The beads were added to the samples and incubated at RT for 45 min with rotation. After capture, the beads were washed three times with 200 μL of 2 × SSC/0.5% (w/v) SDS buffer, twice with 1 × B&W Buffer, and once with 100 μL of Buffer EB.

Step8. Library Construction

75 μL of ddH_2O, 10 μL of 10 × T4 DNA Ligase Buffer, 5 μL of 10 mM dNTP (NEB#N0447L), 5 μL of T4 Polynucleotide Kinase, 4 μL of T4 DNA Polymerase (NEB#M0203L) and 1 μL of Large (Klenow) Fragment were added to the DNAs-bound Dynabeads. The mixture was incubated at 37°C for 30 min with shaking for end repair. The samples were washed twice by adding 200 μL of Tween Wash Buffer (5 mM Tris-HCl (pH 7.5); 500 μM EDTA; 1 M NaCl, 0.05% Tween 20) and incubated at 55°C for 2 min with shaking. Then, 80 μL of ddH_2O, 10 μL of 10 × NEB Buffer 2 (NEB#B7002S), 5 μL of 10 mM dATP and 5 μL of Klenow Fragment (3'→5' exo-) (NEB#M02012L) were added to the DNAs-bound Dynabeads and incubated at 37°C for 30 min with shaking for A-tailing. The samples were washed twice by adding 200 μL of Tween Wash Buffer and incubated at 55°C for 2 min with shaking. Then, 7.6 μL of ddH_2O, 10 μL of 2 × Quick Ligase Buffer, 2 μL of Quick Ligase (NEB#M2200S) and 0.4 μL of 20 μM Y-Adaptor were added to the DNAs-bound Dynabeads and incubated

at RT for 45 min with rotation for sequencing adaptor ligation. After that, the samples were washed twice by adding 200 μL of Tween Wash Buffer and incubated at 55°C for 2 min with shaking. Lastly, the beads were washed in 100 μL of Buffer EB before PCR amplification.

Step9. PCR Amplification

The beads were resuspended in 1 μL of TruePrep Amplify Enzyme (Vazyme Biotech, Cat#TD601), 10 μL of 5 × TAB, 1 μL of i5 Primer, 1 μL of i7 Primer, 2.5 μL of NTR-F, and 2.5 μL of NTR-R and 32 μL of ddH_2O. The following PCR program was performed: 72°C for 5 min, 98°C for 30 s, followed by cycles of 98°C for 10 s, 63°C for 30 s, and 72°C for 1 min. The final cycle number can be estimated by qPCR. After PCR, the libraries were placed on a magnet and eluted into new tubes, then purified for sequencing. A 2% agarose gel was prepared, and the samples were separated by electrophoresis in TAE Buffer. Fragments of 200-800 bp were excised from the gel, and the DNA was recovered using the NucleoSpin Gel and PCR Clean-up kit (Macherey-nagel#740609.50), followed by high-throughput sequencing.

IV: Supplementary Tables

Supplementary Table 1. shRNA sequences used in this study.

pLKO.1-shChd4 1	Forward (5'-3')	CCGG Gactacgacctgttcaagcag CTCGAG ctgcttgaacaggtcgtagtC TTTTTG
	Reverse (5'-3')	AATTCAAAAA Gactacgacctgttcaagcag CTCGAG ctgcttgaacaggtcgtagtC
pLKO.1-shChd4 2	Forward (5'-3')	CCGG gaacgtggtgatggatcttaa CTCGAG ttaagatccatcaccacgttc TTTTTG
	Reverse (5'-3')	AATTCAAAAA gaacgtggtgatggatcttaa CTCGAG ttaagatccatcaccacgttc
pLKO.1-shChd8 1	Forward (5'-3')	CCGG TGCCTGGAAGAAATTGGAGCTCGAGCTCCAATTTCTTCCAGGCATTTTTG
	Reverse (5'-3')	AATTCAAAAA TGCCTGGAAGAAATTGGAG CTCGAG CTCCAATTTCTTCCAGGCA
pLKO.1-shChd8 2	Forward (5'-3')	CCGG GATGTAACTGGTCCAATAAA CTCGAG TTTATTGGACCAGTTACATC TTTTTG
	Reverse (5'-3')	AATTCAAAAA GATGTAACTGGTCCAATAAA CTCGAG TTTATTGGACCAGTTACATC

Supplementary Table 2. sgRNA sequences used in this study.

sgRNA for genome editing	Oligo sequences (5'- to -3')
Ppa2-CTCF motif deletion-1	ctcgctaacaagttcagcag
Ppa2-CTCF motif deletion-2	catcttccaatttccgtgct
Zbtb39-CTCF motif deletion-1	gttcccagaaaccacatagc
Zbtb39-CTCF motif deletion-2	ttgtctgcaactccaattcc
Chd4 knockin-1	ggaaggtcactgctgagtgg
Chd4 knockin-2	tgaggaaggtcactgctgag
Chd8 knockin-1	GCCCCATATAGACCCGTGAT
Chd8 knockin-2	catatagacccgtgatgggt
Smarac4 knockin-1	ttggctgggacgagcgcctc
Smarac4 knockin-2	gcgcctcggggtcaggactc
Smarac5 knockin-1	ttctaatagtttcatttgtg
Smarac5 knockin-2	agtttcatttgtgaggattg
H2-M5-Promoter deletion-1	tatgtctagggtttcccaag
H2-M5-Promoter deletion-2	aaatgcatgaaaatatcacg
Rnf146-Promoter deletion-1	tgtgccggttaaaatgaaca

Rnf146-Promoter deletion-2	cggcttcgttggctgcgagg
Sema4b-Enhancer deletion-1	gtcttcgtaagataggcacg
Sema4b-Enhancer deletion-2	agccagaaactagccgggtg
Chd2-Enhancer deletion-1	ccctgtctcgaaaagctggg
Chd2-Enhancer deletion-2	accggtcaggaatgtctacg

Supplementary Table 3. Primer sequences used for ChIP-qPCR.

Name	Forward primer (5'-3')	Reverse primer (5'-3')
ChIP-qPCR-Ppa2	gagggcgacgtggaatgg	ctccggcttcgtgaactcta
ChIP-qPCR-Zbtb39	aaacgccatcatgctgggaa	cagcttccatttcctccaggt
ChIP-qPCR-Negative	gttggccaggattccctgaa	aagcccccaccatcaatgag

Supplementary Table 4. Antibody information and catalog number.

		Western Blot		
**Primary Antibody**	**Host**	**Manufacturer**	**Catalog Number**	**Dilution**
CTCF	Mouse	Abcam	ab37477	1/1000
HA-tag	Mouse	Abcam	ab9110	1/1000
RAD21	Rabbit	GeneTex	GTX106012	1/1000
Streptavidin (HRP)		Abcam	ab7403	1/1000
β-Actin	Mouse	Sigma	A2228	1/5000
**Secondary Antibody**	**Host**	**Manufacturer**	**Catalog Number**	**Dilution**
HRP Anti-Mouse IgG H&L	Goat	KangChen	KC-MM-035	1/5000
HRP Anti-Rabbit IgG H&L	Goat	KangChen	KC-RB-035	1/5000
		ChIP and HiChIP		
**Primary Antibody**	**Host**	**Manufacturer**	**Catalog Number**	**Dilution**
CTCF	Rabbit	Active motif	61311	——
SMC1A	Rabbit	Bethyl Laboratories	A300-055A	——
IgG	Rabbit	Abcam	ab37415	——
RAD21	Rabbit	Abcam	ab217678	——
H3K27ac	Rabbit	Active motif	39133	——

Supplementary Table 5. Primer sequences used for RT-qPCR.

Name	Forward primer (5'-3')	Reverse primer (5'-3')
mPpa2	cgcgtcatgtccctgtacc	aatgtcgtggaaaggggagat
mZbtb39	cgtggtggacttcattacccc	gcgttcagcgacctcataga
mCtcf	gatcctacccttctccagatgaa	gtaccgtcacaggaacaggt
mGapdh	aactttggcattgtggaagggctca	ttggcagcaccagtggatgcaggga
mChd2	AGGAGGTCAAATCGAAGCAGA	GCCTCTTCTTTTCGGACTCCC
mSema4b	CAAGACGCTGTATGTGGGGG	TTGACAGTCACGCTTTGGGTC
mRnf146	ATGCCAGTAGTGATAGTGAGGAT	CCCCTGCAACTGATCGGTC
mEchdc1	TGACGCTAAACAACCCCAATAAA	GTGCCTTCACAGCATTCAGAT
mH2-M5	TGAAGGGCTGAACCCAGGAA	AGGGAAGTAAAATGGGAAAAGTGA
mZfp57	ATGGCAGCTAGGAAACAGTCT	TGGTAAAGGGTCTTCTGTGTAGA
mChd4	GAAATTGCTGCGGCACCATTA	AGCCATCATTGTAGTTGACCTG
mChd8	ACTGGTACACCCCTTCGAC	AGGAGCTGAACCCTTTACTGG

Supplementary Table 6. Mapping statistic and peak calling information for CTCF and SMC1 ChIP-seq.

Sample name	Total reads	Trimmed reads	Mapping ratio (%)	Unique reads (mm10)	Number of peaks (mm10)	scale factor
0h_CTCF	26231407	26198896	89.70%	13979230	29934	1
0h_SMC1	26849038	26842440	93.20%	14079264	16724	1
0h_input	31981344	31974676	92.10%	17247678	-	-
12h_CTCF	27281241	27257975	89.20%	14161456	2248	0.79
12h_SMC1	31310657	31294559	93.20%	17308204	10	1.42
12h_input	42883397	42873625	91.70%	23175485	-	-

Supplementary Table 7. Mapping statistic and peak calling information for BIOTIN ChIP-seq.

Sample name	Total reads	Trimmed reads	Mapping ratio	Unique reads	Subsampled reads	Peak num	Scale factor
CHD4_0h_rep3	45368422	45366217	94.89%	31531151	-	111947	0.989
CHD4_0h_rep4	59593837	59591358	95.45%	41132821	30000000	187294	0.884
CHD4_0h_input	90562921	90560364	98.06%	63907538	-	-	-
CHD4_12h_rep1	40985710	40984367	96.18%	26360480	-	105001	1.111
CHD4_12h_rep4	62313541	62310806	95.42%	40194932	30000000	159918	0.999
CHD4_12h_input	100002677	100000032	98.93%	67300658	-	-	-

CHD8_0h_rep2	59253695	59251472	68.66%	22076709	-	51943	0.869
CHD8_0h_rep3	47639462	47637772	79.43%	20511655	-	40880	1.203
CHD8_0h_input	62675052	62673360	98.96%	38761600	-	-	-
CHD8_12h_rep3	56413657	56411443	71.73%	23193053	-	51840	0.825
CHD8_12h_rep4	49823698	49821784	85.51%	22627102	-	42297	1.14
CHD8_12h_input	61168682	61166870	98.93%	35626305	-	-	-
smarca4_0h_rep1	48093698	48092191	95.92%	31062419	-	92456	0.875
smarca4_0h_rep2	36721089	36719816	96.59%	25064246	-	70428	1.286
smarca4_0h_input	40348483	40347512	98.30%	28286699	-	-	-
smarca4_12h_rep1	42047883	42046518	96.01%	28155033	-	96364	0.957
smarca4_12h_rep2	40932520	40931030	96.42%	29690741	-	102636	0.898
smarca4_12h_input	44359722	44358530	99.06%	31864922	-	-	-
smarca5_0h_rep1	35830257	35829056	95.29%	24642524	-	55488	1.177
smarca5_0h_rep2	41529209	41527496	95.79%	29848951	-	73238	0.932
smarca5_0h_input	45399547	45398692	98.99%	32816231	-	-	-
smarca5_12h_rep1	35588023	35586467	94.89%	25479795	-	71067	1.036
smarca5_12h_rep2	41149682	41147990	97.13%	28338484	-	67059	0.857
smarca5_12h_input		56153579	99.02%	40440053	-	-	-

Supplementary Table 8. Primer sequences used for MNase-4C.

Primer name	Primer sequences (5'- to -3')	Chr	Start	END	strand
Biotin-Pcdh-se-f-F	5' biotin-ctccatgtgccatctggtgg	chr18	37883720	37883739	+
P5-Pcdh-se-f-F	aatgatacggcgaccaccgagatctacactaatctta acactctttccctacacgacgctcttccgatctggagcactgttgcttaga gattctc	chr18	37883751	37883775	+
Biotin-aglobin-F1	5' biotin-gggttttggaaaaagcagcc	chr11	32238467	32238486	+
Biotin-aglobin-F2	5' biotin-taggcctctgctaccctctg	chr11	32238645	32238664	+
Biotin-aglobin-F3	5' biotin-ttctctctgagcccaaaggg	chr11	32238823	32238842	+
Biotin-aglobin-F4	5' biotin-cagagggtagcagaggccta	chr11	32238645	32238664	-
P5-aglobin-F1	aatgatacggcgaccaccgagatctacactctttccctacacgacgct cttccgatctaaaacctccagtgatgccaaccc	chr11	32238505	32238527	+
P5-aglobin-F2	aatgatacggcgaccaccgagatctacactctttccctacacgacgct cttccgatcttcagggtgcatagcactaccagg	chr11	32238671	32238693	+
P5-aglobin-F3	aatgatacggcgaccaccgagatctacactctttccctacacgacgct cttccgatcttgccacacacacttgccaagggg	chr11	32238844	32238866	+

P5-aglobin-F4	aatgatacggcgaccaccgagatctacactctttccctacacgacgct cttccgatctcctcagtttgctcaaaggccagc	chr11	32238615	32238637	-
Biotin-malt1-F1	5’ biotin-aattggctggagcagccaccaggtg	chr18	65300855	65300879	+
Biotin-malt1-F2	5’ biotin-agccaccctgagtgcagtcaggact	chr18	65489977	65490001	+
Biotin-malt1-F3	5’ biotin-cgactcctacagctgtcctccgatc	chr18	65489869	65489893	-
Biotin-malt1-F4	5’ biotin-tctctagacgggttaaacacacaca	chr18	65490215	65490239	-
P5-malt1-F1	aatgatacggcgaccaccgagatctacactatagcct acactctttccctacacgacgctcttccgatctagccaccaggtggcag gataggcat	chr18	65300868	65300892	+
P5-malt1-F2	aatgatacggcgaccaccgagatctacactatagcct acactctttccctacacgacgctcttccgatctctgctggcacacatgaa gtaactgt	chr18	65490026	65490050	+
P5-malt1-F3	aatgatacggcgaccaccgagatctacacatagaggcacactctttcc ctacacgacgctcttccgatctatgcctatggtggtatgcattgacc	chr18	65489839	65489863	-
P5-malt1-F4	aatgatacggcgaccaccgagatctacaccctatcctacactctttccc tacacgacgctcttccgatctcactgttaatgtctcttacacacac	chr18	65490179	65490203	-
Biotin-Sox2-F1	5’ biotin-tactagttggacagtcgccc	chr3	34649576	34649595	+
Biotin-Sox2-F2	5’ biotin-gagagtgtttgcaaaaaggg	chr3	34650051	34650070	+
Biotin-Sox2-F3	5’ biotin-catcccaattgcacttcgcc	chr3	34650222	34650241	+
Biotin-Sox2-F4	5’ biotin-cccttttttgcaaacactctc	chr3	34650051	34650070	-
P5-Sox2-F1	aatgatacggcgaccaccgagatctacactctttccctacacgacgct cttccgatctaaccacccatgggccttgcccca	chr3	34649598	34649620	+
P5-Sox2-F2	aatgatacggcgaccaccgagatctacactctttccctacacgacgct cttccgatctgcctctttaagactagggctggg	chr3	34650085	34650107	+
P5-Sox2-F3	aatgatacggcgaccaccgagatctacactctttccctacacgacgct cttccgatcttccgcttccccccaactattctc	chr3	34650252	34650274	+
P5-Sox2-F4	aatgatacggcgaccaccgagatctacactctttccctacacgacgct cttccgatctctgccttgacaactcctgatact	chr3	34650024	34650046	-
Biotin-Klf4-F1	5’ biotin-gtgcgcggagtttgtttatttagc	chr4	55532631	55532654	-
Biotin-Klf4-F2	5’ biotin-gagcaagcgagcgagaagttataag	chr4	55532501	55532525	-
Biotin-Klf4-F3	5’ biotin-gccgccgcgcgcttcttacttataa	chr4	55532483	55532507	+
Biotin-Klf4-F4	5’ biotin-agttccgcgccaccgcggccattct	chr4	55532245	55532269	-
P5-Klf4-F1	aatgatacggcgaccaccgagatctacactatagcctacactctttccc tacacgacgctcttccgatctttagctaccatggcaacgcgcagtg	chr4	55532611	55532635	-
P5-Klf4-F2	aatgatacggcgaccaccgagatctacactatagcctacactctttccc tacacgacgctcttccgatctgttataagtaagaagcgcgcggcg	chr4	55532485	55532508	-
P5-Klf4-F3	aatgatacggcgaccaccgagatctacactatagcctacactctttccc tacacgacgctcttccgatctataacttctcgctcgcttgctcccc	chr4	55532504	55532528	+
P5-Klf4-F4	aatgatacggcgaccaccgagatctacactatagcctacactctttccc	chr4	55532226	55532249	-

	tacacgacgctcttccgatct attctcacctggcggcgccgcccg				
Biotin-PAX9-F1	5’ biotin-agtggctgatgggggacgttgtcag	chr12	56695868	56695892	+
Biotin-PAX9-F2	5’ biotin-gccactgacccttggagggtgctta	chr12	56695989	56696013	-
Biotin-PAX9-F3	5’ biotin-gcttcgcatcgtggaattagcccaa	chr12	56696647	56696671	+
Biotin-PAX9-F4	5’ biotin-ccgcgctgccctacaaccacattta	chr12	56697024	56697048	+
P5-PAX9-F1	aatgatacggcgaccaccgagatctacacggctctgaacactctttcc ctacacgacgctcttccgatct tgtattgactcagactgcgtccgtc	chr12	56695958	56695982	+
P5-PAX9-F2	aatgatacggcgaccaccgagatctacacaggcgaagacactctttc cctacacgacgctcttccgatct acgcagtctgagtcaatacagcgcg	chr12	56695953	56695977	-
P5-PAX9-F3	aatgatacggcgaccaccgagatctacacggctctgaacactctttcc ctacacgacgctcttccgatctttgtgacatcagccgccagctacgg	chr12	56696686	56696710	+
P5-PAX9-F4	aatgatacggcgaccaccgagatctacacggctctgaacactctttcc ctacacgacgctcttccgatctatcacggcggcagcagctaaggt	chr12	56697065	56697087	+
Biotin-Nkx2-1-F 1	5’ biotin-ttcctcctccagccgacgccgaatc	chr12	56535061	56535085	-
Biotin-Nkx2-1-F 2	5’ biotin-taaaacaaacgcgaggcagcccccg	chr12	56535247	56535271	-
Biotin-Nkx2-1-F 3	5’ biotin-ggtaacaccagaatatttggcaaaggg	chr12	56535147	56535173	-
Biotin-Nkx2-1-F 4	5’ biotin-cgctgttccgcatggtgtcctggta	chr12	56534739	56534763	+
P5-Nkx2-1-F1	aatgatacggcgaccaccgagatctacactatagcctacactctttccc tacacgacgctcttccgatctcaaagcacacgactccgttctcagt	chr12	56535023	56535047	-
P5-Nkx2-1-F2	aatgatacggcgaccaccgagatctacactatagcctacactctttccc tacacgacgctcttccgatctagccaattaaggcggactcggtcca	chr12	56535210	56535234	-
P5-Nkx2-1-F3	aatgatacggcgaccaccgagatctacactatagcctacactctttccc tacacgacgctcttccgatctaaaagtagcgaggcttcgccttccc	chr12	56535115	56535139	-
P5-Nkx2-1-F4	aatgatacggcgaccaccgagatctacactatagcctacactctttccc tacacgacgctcttccgatct tcatgttgcccaggttgccgttgca	chr12	56534778	56534802	+
Biotin-Deptor-F	5’ biotin-ttctcctgcttacccgcagggatt	chr15	55112330	55112353	+
P5-Deptor-F	aatgatacggcgaccaccgagatctacac cctatcct acactctttccctacacgacgctcttccgatct accagccaatcgcgtctgagcca	chr15	55112359	55112381	+
Biotin-Dusp57-F	5’ biotin-aaagactcctctgtttggtctac	chr1	166125338	166125360	+
P5- Dusp57-F	aatgatacggcgaccaccgagatctacac gtactgac acactctttccctacacgacgctcttccgatct gactgccactccctatccacacc	chr1	166125365	166125387	+
Biotin-Dppa3-F	5’ biotin-ccccattcacagactgactgctaa	chr6	122626416	122626439	+
P5-Dppa3-F	aatgatacggcgaccaccgagatctacac	chr6	12262644	122626466	+

	cctatcctacactctttccctacacgacgctcttccgatctgggtcttggttt taggtcttttcaa		2		
Biotin-Eif2b5-F	gatctcacccctctggggtgcatac	chr16	20498693	20498717	+
P5-Eif2b5-F	aatgatacggcgaccaccgagatctacac caggacgtacactctttccctacacgacgctcttccgatcttcccagtag cacctactgcggtcag	chr16	20498721	20498745	+
Biotin-Exd1-F	5' biotin-aggcgctaacgtcaaagcttggc	chr2	119547632	119547654	-
P5-Exd1-F	aatgatacggcgaccaccgagatctacac aggcgaagacactctttccctacacgacgctcttccgatcttctcgagc ccggaatgaatcagcc	chr2	119547608	119547631	-
Biotin-Tmem14a-F	5' biotin-aaccagctccagagaggactcagct	chr1	21218736	21218760	+
P5-Tmem14a-F	aatgatacggcgaccaccgagatctacac cctatcct acactctttccctacacgacgctcttccgatctctgtgcactctgctctcta aggcg	chr1	21218761	21218784	+
Biotin-Ppa2-CTCF-F	5' biotin-acaagttcagcagagggcgctctttc	chr3	133552168	133552193	-
P5-Ppa2-CTCF-F	aatgatacggcgaccaccgagatctacaccctatcctacactctttccc tacacgacgctcttccgatctatcttccaatttccgtgctaggcgg	chr3	133552142	133552166	-

Supplementary Table 9. Statistic information of M4C data.

Sample name	Total reads	Trimmed reads	Reads containing primer sequence	Mapped reads	Unique reads	Reads in cis	Selected reads
globin_p1	2480645	10716 (0.43%)	2,469,928	2365712	15649	12131	4806
globin_p2	809465	160040 (19.77%)	649,425	478832	10301	8463	2017
globin_p3	578380	36696 (6.34%)	541,684	529429	4803	4007	1545
globin_p4	660601	50868 (7.70%)	609,733	569874	7706	6871	1738
malt1_F1	4078555	100109 (2.45%)	3,965,538	3846771	25595	17910	9064
malt1_F2	30875591	506658 (1.64%)	30,123,914	23063336	1208148	60961	31868
malt1_F3	3725154	627573 (16.85%)	3,053,875	2581590	18619	9006	4429
malt1_F4	3137261	70625 (2.25%)	3,038,965	1039131	606389	478604	7159
pcdh	3774343	175789 (4.66%)	3,011,405	2827964	36087	28412	12600
klf4_F1	12025223	16210 (0.13%)	11,936,262	7935954	17066	12083	7253
klf4_F2	2121797	354055 (16.69%)	1,726,151	1344231	13653	9912	4771
klf4_F3	5946750	283590 (4.77%)	5,344,845	3387684	17742	13161	6217
klf4_F4	2857409	59192 (2.07%)	2,626,514	2612986	10346	8377	4617
sox2_F1	5455629	236016 (4.33%)	5,214,869	3902041	87625	59590	28323

sox2_F2	145046	7258 (5.00%)	117,748	111847	7331	5688	2996
sox2_F3	625783	11466 (1.83%)	602,459	513078	14829	10275	5797
sox2_F4	656087	29537 (4.50%)	588,338	536598	42448	30420	15238
nkx21_F1	10744455	45780 (0.43%)	10,552,439	7825140	32396	19940	9079
nkx21_F2	11782890	51737 (0.44%)	11,688,176	9862812	28078	14847	7509
nkx21_F3	4532415	24886 (0.55%)	4,139,273	3966670	32528	24643	11079
nkx21_F4	4837272	18013 (0.37%)	4,778,014	4122332	16266	10060	4543
pax9_F1	7245127	71613 (0.99%)	1,779,740	1570464	7804	5970	2620
pax9_F2	12580099	107721 (0.86%)	5,452,948	4862306	8855	5774	2860
pax9_F3	1531461	41420 (2.70%)	1,481,409	1365979	19939	16147	8719
pax9_F4	16660388	143625 (0.86%)	15,140,812	8524718	32030	20087	10100
0h_Chd2_rep1	13971949	2279 (0.02%)	13,941,873	8719512	28171	19295	8517
0h_Chd2_rep2	18173187	7121 (0.04%)	18,094,514	13009920	23622	17285	7931
0h_Deptor_rep1	3923329	918 (0.02%)	3,856,199	3794103	25514	19133	8679
0h_Deptor_rep2	3013665	994 (0.03%)	2,964,739	2916043	21293	16141	7511
0h_Dppa3_rep1	5267795	18331 (0.35%)	5,235,232	1073415	9529	6242	3199
0h_Dppa3_rep2	6845921	13876 (0.20%)	6,823,930	853583	20530	9699	5056
0h_Dusp27_rep1	8421696	8952 (0.11%)	6,405,826	5888378	20710	15642	6661
0h_Dusp27_rep2	777457	445 (0.06%)	644,503	610784	11017	8476	3866
0h_Eif2b5_rep1	500143	437 (0.09%)	494,199	490691	8058	6311	3231
0h_Eif2b5_rep2	450265	449 (0.10%)	443,830	441108	6786	5327	2827
0h_Exd1_rep1	17086985	3212 (0.02%)	16,930,790	13004315	31985	21038	12323
0h_Exd1_rep2	16852047	22723 (0.13%)	16,636,276	11960917	27024	17612	10486
0h_Sema4b_rep1	5569585	2938 (0.05%)	5,364,492	4734926	39395	28285	13108
0h_Sema4b_rep2	4591915	4669 (0.10%)	4,386,365	3949643	26135	19003	7523
0h_Tmem14a_rep1	6706162	32183 (0.48%)	6,048,770	4754700	16643	12540	6721
0h_Tmem14a_rep2	7908718	20104 (0.25%)	7,464,250	5010229	18156	13573	7258
0h_ppa2	3516485	89974 (2.56%)	3,409,000	2913049	31590	7402	5966
12h_Chd2_rep1	16301353	10665 (0.07%)	16,249,046	13282878	19066	14005	6466
12h_Chd2_rep2	15207395	7122 (0.05%)	15,163,671	12333421	18399	13847	6391
12h_Deptor_rep1	4374539	2090 (0.05%)	4,168,954	4118464	23256	17509	8061
12h_Deptor_rep2	5575313	2412 (0.04%)	5,311,739	5251173	22619	17049	7606
12h_Dppa3_rep1	9498050	22042 (0.23%)	9,462,403	595062	10881	7905	3684
12h_Dppa3_rep2	4652910	18838 (0.40%)	4,626,749	820298	13450	7793	3974
12h_Dusp27_rep1	3798370	1482 (0.04%)	3,595,720	3349102	21201	15612	7527
12h_Dusp27_rep2	4769503	2512 (0.05%)	4,599,151	4374504	22266	16344	7876
12h_Eif2b5_rep1	412105	1215 (0.29%)	408,825	406686	5240	4164	2222
12h_Eif2b5_rep2	581608	622 (0.11%)	576,854	574585	5574	4405	2294
12h_Exd1_rep1	11755958	3668 (0.03%)	11,615,918	8408653	25439	17249	10055
12h_Exd1_rep2	30478812	7580 (0.02%)	30,253,719	25296676	26298	15260	9464
12h_Sema4b_rep1	2345407	1881 (0.08%)	2,170,666	2000087	19707	15163	5757
12h_Sema4b_rep2	3455428	14962 (0.43%)	3,304,126	2883032	23769	17974	8113
12h_Tmem14a_rep1	8124299	1489 (0.02%)	7,674,743	6665015	22049	17035	8113

12h_Tmem14a_rep 2	6466162	1461 (0.02%)	6,061,571	5489969	26675	21130	9811
12h_ppa2	3143751	767987 (24.43%)	2,366,414	2065973	18866	5246	3991

Supplementary Table 10. Statistic and quality control information of CTCF MNase HiChIP data.

Type	CTCF_rep1		CTCF_rep2		merge	
Total Read Pairs	371,734,851	100%	237,253,976	100%		
Unmapped Read Pairs	35,159,940	9.46%	22,312,151	9.40%		
Mapped Read Pairs	289,111,255	77.77%	188,341,436	79.38%		
PCR Dup Read Pairs	168,204,741	45.25%	113,965,924	48.04%		
No-Dup Read Pairs	120,906,514	32.52%	74,375,512	31.35%	195,282,026	100.00%
No-Dup Cis Read Pairs	99,184,516	82.03%	60,651,850	81.55%	159,836,366	81.85%
No-Dup Trans Read Pairs	21,721,998	17.97%	13,723,662	18.45%	35,445,660	18.15%
No-Dup Valid Read Pairs (cis >= 1kb + trans)	64,834,168	53.62%	37,608,812	50.57%	102,442,980	52.46%
No-Dup Cis Read Pairs < 1kb	56,072,346	46.38%	36,766,700	49.43%	92,839,046	47.54%
No-Dup Cis Read Pairs >= 1kb	43,112,170	35.66%	23,885,150	32.11%	66,997,320	34.31%
No-Dup Cis Read Pairs >= 10kb	35,734,621	29.56%	20,143,753	27.08%	55,878,374	28.61%

Supplementary Table 11. Statistic and quality control information of RAD21 MNase HiChIP data.

Type	RAD21_rep1		RAD21_rep2		RAD21_rep3		merge	
Total Read Pairs	92,328,644	100%	56,085,205	100%	61,020,798	100%		
Unmapped Read Pairs	4,841,015	5.24%	5,405,269	9.64%	6,259,132	10.26%		
Mapped Read Pairs	74,067,244	80.22%	44,562,164	79.45%	47,844,295	78.41%		
PCR Dup Read Pairs	64,272,216	69.61%	22,238,683	39.65%	27,639,726	45.30%		
No-Dup Read Pairs	9,795,028	10.61%	22,323,481	39.80%	20,204,569	33.11%	52,323,078	100.00%
No-Dup Cis Read Pairs	8,188,370	83.60%	18,520,768	82.97%	17,116,855	84.72%	43,825,993	83.76%
No-Dup Trans Read Pairs	1,606,658	16.40%	3,802,713	17.03%	3,087,714	15.28%	8,497,085	16.24%
No-Dup Valid Read Pairs (cis >= 1kb + trans)	7,076,913	72.25%	12,650,061	56.67%	11,622,839	57.53%	31,349,813	59.92%
No-Dup Cis Read Pairs < 1kb	2,718,115	27.75%	9,673,420	43.33%	8,581,730	42.47%	20,973,265	40.08%
No-Dup Cis Read Pairs >= 1kb	5,470,255	55.85%	8,847,348	39.63%	8,535,125	42.24%	22,852,728	43.68%
No-Dup Cis Read Pairs >= 10kb	4,575,122	46.71%	7,354,261	32.94%	7,071,735	35.00%	19,001,118	36.31%

Supplementary Table 12. Statistic and quality control information of H3K27ac MNase HiChIP data.

Type	H3K27ac_IAA_0h_rep1		H3K27ac_IAA_0h_rep2		merge	
Total Read Pairs	512063841	100.00%	506378943	100.00%		
Unmapped Read Pairs	57883119	11.30%	57972741	11.45%		
Mapped Read Pairs	387391908	75.65%	383230997	75.68%		

PCR Dup Read Pairs	271921331	53.10%	279240103	55.14%		
No-Dup Read Pairs	115470577	22.55%	103990894	20.54%	219461471	100.00%
No-Dup Cis Read Pairs	99966804	86.57%	89887699	86.44%	189854503	86.51%
No-Dup Trans Read Pairs	15503773	13.43%	14103195	13.56%	29606968	13.49%
No-Dup Valid Read Pairs (cis >= 1kb + trans)	52771958	45.70%	47680962	45.85%	100452920	45.77%
No-Dup Cis Read Pairs < 1kb	62698619	54.30%	56309932	54.15%	119008551	54.23%
No-Dup Cis Read Pairs >= 1kb	37268185	32.28%	33577767	32.29%	70845952	32.28%
No-Dup Cis Read Pairs >= 10kb	28265085	24.48%	25497161	24.52%	53762246	24.50%

**Type**	**H3K27ac_IAA_12h_rep1**		**H3K27ac_IAA_12h_rep2**		**merge**	
Total Read Pairs	504082700	100.00%	502838321	100.00%		
Unmapped Read Pairs	54684769	10.85%	58739356	11.68%		
Mapped Read Pairs	379665301	75.32%	379681285	75.51%		
PCR Dup Read Pairs	241467147	47.90%	238518676	47.43%		
No-Dup Read Pairs	138198154	27.42%	141162609	28.07%	279360763	100.00%
No-Dup Cis Read Pairs	119803183	86.69%	122703622	86.92%	242506805	86.81%
No-Dup Trans Read Pairs	18394971	13.31%	18458987	13.08%	36853958	13.19%
No-Dup Valid Read Pairs (cis >= 1kb + trans)	62647697	45.33%	62137038	44.02%	124784735	44.67%
No-Dup Cis Read Pairs < 1kb	75550457	54.67%	79025571	55.98%	154576028	55.33%
No-Dup Cis Read Pairs >= 1kb	44252726	32.02%	43678051	30.94%	87930777	31.48%
No-Dup Cis Read Pairs >= 10kb	33377656	24.15%	32960248	23.35%	66337904	23.75%

References

Bhattacharyya, S., Chandra, V., Vijayanand, P., and Ay, F. (2019). Identification of significant chromatin contacts from HiChIP data by FitHiChIP. Nat Commun 10, 4221.

Dobin, A., Davis, C.A., Schlesinger, F., Drenkow, J., Zaleski, C., Jha, S., Batut, P., Chaisson, M., and Gingeras, T.R. (2013). STAR: ultrafast universal RNA-seq aligner. Bioinformatics 29, 15-21.

Durand, N.C., Shamim, M.S., Machol, I., Rao, S.S., Huntley, M.H., Lander, E.S., and Aiden, E.L. (2016). Juicer Provides a One-Click System for Analyzing Loop-Resolution Hi-C Experiments. Cell Syst 3, 95-98.

Ernst, J., and Kellis, M. (2012). ChromHMM: automating chromatin-state discovery and characterization. Nat Methods 9, 215-216.

Grant, C.E., Bailey, T.L., and Noble, W.S. (2011). FIMO: scanning for occurrences of a given motif. Bioinformatics 27, 1017-1018.

Harmston, N., Ing-Simmons, E., Perry, M., Baresic, A., and Lenhard, B. (2015). GenomicInteractions: An R/Bioconductor package for manipulating and investigating chromatin interaction data. BMC Genomics 16, 963.

Hu, S., Peng, L., Song, A., Ji, Y.X., Cheng, J., Wang, M., and Chen, F.X. (2023). INTAC endonuclease and phosphatase modules differentially regulate transcription by RNA polymerase II. Mol Cell 83, 1588-1604 e1585.

Jia, Z., Li, J., Ge, X., Wu, Y., Guo, Y., and Wu, Q. (2020). Tandem CTCF sites function as insulators to balance spatial chromatin contacts and topological enhancer-promoter selection. Genome Biol 21, 75.

Langmead, B., and Salzberg, S.L. (2012). Fast gapped-read alignment with Bowtie 2. Nat Methods 9, 357-359.

Li, B., and Dewey, C.N. (2011). RSEM: accurate transcript quantification from RNA-Seq data with or without a reference genome. BMC Bioinformatics 12, 323.

Li, G., Chen, Y., Snyder, M.P., and Zhang, M.Q. (2017). ChIA-PET2: a versatile and flexible pipeline for ChIA-PET data analysis. Nucleic Acids Res 45, e4.

Liang, Z., Li, G., Wang, Z., Djekidel, M.N., Li, Y., Qian, M.P., Zhang, M.Q., and Chen, Y. (2017). BL-Hi-C is an efficient and sensitive approach for capturing structural and regulatory chromatin interactions. Nat Commun 8, 1622.

Liao, Y., Smyth, G.K., and Shi, W. (2014). featureCounts: an efficient general purpose program for assigning sequence reads to genomic features. Bioinformatics 30, 923-930.

Love, M.I., Huber, W., and Anders, S. (2014). Moderated estimation of fold change and dispersion for RNA-seq data with DESeq2. Genome Biol 15, 550.

Narita, T., Higashijima, Y., Kilic, S., Liebner, T., Walter, J., and Choudhary, C. (2023). Acetylation of histone H2B marks active enhancers and predicts CBP/p300 target genes. Nat Genet 55, 679-692.

Ramirez, F., Ryan, D.P., Gruning, B., Bhardwaj, V., Kilpert, F., Richter, A.S., Heyne, S., Dundar, F., and Manke, T. (2016). deepTools2: a next generation web server for deep-sequencing data analysis. Nucleic Acids Res 44, W160-165.

Robinson, M.D., McCarthy, D.J., and Smyth, G.K. (2010). edgeR: a Bioconductor package for differential expression analysis of digital gene expression data. Bioinformatics 26, 139-140.

Ross-Innes, C.S., Stark, R., Teschendorff, A.E., Holmes, K.A., Ali, H.R., Dunning, M.J., Brown, G.D., Gojis, O., Ellis, I.O., Green, A.R., *et al.* (2012). Differential oestrogen receptor binding is associated with clinical outcome in breast cancer. Nature 481, 389-393.

Servant, N., Varoquaux, N., Lajoie, B.R., Viara, E., Chen, C.J., Vert, J.P., Heard, E., Dekker, J., and Barillot, E. (2015). HiC-Pro: an optimized and flexible pipeline for Hi-C data processing. Genome Biol 16, 259.

Stansfield, J.C., Cresswell, K.G., and Dozmorov, M.G. (2019). multiHiCcompare: joint normalization and comparative analysis of complex Hi-C experiments. Bioinformatics 35, 2916-2923.

Yang, T., Zhang, F., Yardimci, G.G., Song, F., Hardison, R.C., Noble, W.S., Yue, F., and Li, Q. (2017). HiCRep: assessing the reproducibility of Hi-C data using a stratum-adjusted correlation coefficient. Genome Res 27, 1939-1949.

Yu, G., Wang, L.G., and He, Q.Y. (2015). ChIPseeker: an R/Bioconductor package for ChIP peak annotation, comparison and visualization. Bioinformatics 31, 2382-2383.

Zhang, J., Hu, G., Lu, Y., Ren, H., Huang, Y., Wen, Y., Ji, B., Wang, D., Wang, H., Liu, H., *et al.* (2024). CTCF mutation at R567 causes developmental disorders via 3D genome rearrangement and abnormal neurodevelopment. Nat Commun 15, 5524.

Zhang, Y., Liu, T., Meyer, C.A., Eeckhoute, J., Johnson, D.S., Bernstein, B.E., Nusbaum, C., Myers, R.M., Brown, M., Li, W., *et al.* (2008). Model-based analysis of ChIP-Seq (MACS). Genome Biol 9, R137.
